# Effectiveness of Mycophenolate Mofetil Among Patients With Progressive IgA Nephropathy: A Randomized Clinical Trial

**DOI:** 10.1001/jamanetworkopen.2022.54054

**Published:** 2023-02-06

**Authors:** Fan Fan Hou, Di Xie, Jun Wang, Xin Xu, Xiaobing Yang, Jun Ai, Sheng Nie, Min Liang, Guobao Wang, Nan Jia

**Affiliations:** 1 Division of Nephrology, Nanfang Hospital, Southern Medical University, Guangzhou, Guangdong, China; 2 National Clinical Research Center for Kidney Disease, Guangzhou, Guangdong, China; 3 State Key Laboratory of Organ Failure Research, Guangzhou, Guangdong, China; 4 Guangdong Provincial Institute of Nephrology, Guangzhou, Guangdong, China; 5 Guangdong Provincial Key Laboratory of Renal Failure Research, Guangzhou, Guangdong, China

## Abstract

**Importance:**

The role of mycophenolate mofetil (MMF) in management of immunoglobulin A nephropathy (IgAN) remains highly controversial.

**Objective:**

To evaluate the efficacy and safety of MMF in patients with IgAN at high risk of kidney function loss.

**Design, Setting, and Participants:**

This randomized clinical trial with open-label, blinded end-point design was conducted among adults with IgAN, proteinuria greater than 1.0 g/d, and estimated glomerular filtration rate (eGFR) greater than 30 and less than 60 mL/min/1.73m^2^ or with persistent hypertension from September 2013 to December 2015. During a 3-month run-in period, 238 patients received optimized supportive care (SC), including losartan. Patients with a urinary protein excretion rate of 0.75 g/d or greater despite of 3 months optimized SC were enrolled into the trial for 3 years. Survivors of the trial who did not receive dialysis or transplant were followed up after the trial for a median (IQR) of 60 (47-76) months. Data were analyzed from March through June 2022.

**Interventions:**

A total of 170 participants were randomized in a 1:1 ratio to receive MMF (initially, 1.5 g/d for 12 months, maintained at 0.75-1.0 g for at least 6 months) plus SC or SC alone.

**Main Outcomes and Measures:**

The primary outcomes were (1) a composite of doubling of serum creatinine, end-stage kidney disease (dialysis, transplant, or kidney failure without receiving kidney replacement therapy), or death due to kidney or cardiovascular cause and (2) progression of chronic kidney disease.

**Results:**

Among 170 randomized patients (mean [SD] age 36.6 [9.4] years; 94 [55.3%] male patients), 85 patients received MMF with SC and 85 patients received SC alone. The mean (SD) eGFR was 50.1 (17.9) mL/min/1.73m^2^ and mean (SD) proteinuria level was 1.9 (1.7) g/d; 168 patients (98.8%) completed the trial, and 157 participants (92.4%) survived and did not receive dialysis or transplant. Primary composite outcome events occurred in 6 patients (7.1%) in the MMF group and 18 patients (21.2%) in the SC group (adjusted hazard ratio [aHR], 0.23; 95% CI, 0.09-0.63). Progression of chronic kidney disease occurred in 7 participants (8.2%) in the MMF group and 23 participants (27.1%) in the SC group (aHR, 0.23; 95% CI, 0.10-0.57). The effect of MMF treatment on primary outcomes was consistent across prespecified subgroups, with no significant interaction per subgroup. During posttrial follow-up, annual loss of eGFR accelerated after discontinuation of MMF; mean (SD) annual eGFR loss during the study period was 2.9 (1.0) mL/min/1.73m^2^ in the MMF group and 6.1 (1.2) mL/min/1.73m^2^ among 66 patients in the MMF group who discontinued MMF after the trial. Serious adverse events were not more frequent with MMF vs SC alone.

**Conclusions and Relevance:**

This study found that addition of MMF to SC compared with SC alone significantly reduced risk of disease progression among patients with progressive IgAN.

**Trial Registration:**

ClinicalTrials.gov Identifier: NCT01854814

## Key Points

**Question:**

Is mycophenolate mofetil (MMF) effective in patients with immunoglobulin A (IgA) nephropathy at high risk of kidney function loss treated with optimized supportive care?

**Findings:**

In this randomized clinical trial including 170 participants with IgA nephropathy, the addition of MMF to optimized supportive care significantly reduced risk of a composite outcome (creatinine doubling, indication for kidney replacement therapy, or death due to kidney or cardiovascular cause) and progression of chronic kidney disease.

**Meaning:**

These findings suggest that MMF may be beneficial in patients with progressive IgA nephropathy.

## Teaser

This randomized clinical trial investigates the risk of kidney outcomes among patients with progressive IgA nephropathy receiving mycophenolate mofetil with supportive care vs supportive care alone.

## Introduction

Immunoglobulin A nephropathy (IgAN) is the most common type of glomerulonephritis.^1^ Up to 40% of patients with IgAN progress to end-stage kidney disease (ESKD) over 10 to 20 years, making it the leading cause of kidney failure in many parts of the world.^2^

The appropriate therapy of IgAN remains uncertain. The central role of immune and autoimmune activation in the pathogenesis of IgAN^3,4,5,6^ suggests a potential benefit of immunosuppression for treating the disease. However, the key randomized clinical trials (RCTs) on the efficacy of immunosuppression in IgAN have yielded conflicting results.^7,8,9,10,11,12^ The Supportive vs Immunosuppressive Therapy for the Treatment of Progressive IgAN (STOP-IgAN) trial^8^ found that addition of immunosuppression to intensive supportive care (SC) did not improve the clinical outcome. However, the Therapeutic Evaluation of Steroid in IgA Nephropathy Global (TESTING) study^7,13^ reported that steroid therapy significantly reduced the frequency of primary kidney outcomes with increased adverse events. Given that most reported RCTs are small and not sufficiently powered for evaluating the efficacy of immunosuppression in IgAN, it remains unanswered whether patients with IgAN would benefit from immunosuppressive therapy.

Current management of IgAN remains focused on non–immunosuppressive-based strategies, known as SC, to reduce proteinuria and slow disease progression. This encompasses optimal inhibition of the renin-angiotensin system (RAS), rigorous blood pressure control, and lifestyle modification.^14^ However, despite intensive SC, a considerable number of patients still have massive proteinuria and remain at high risk of disease progression. The Kidney Disease Improving Global Outcomes (KDIGO) guidelines suggest the use of systemic glucocorticoids in patients who have a proteinuria level greater than 1 g/d and an estimated glomerular filtration rate (eGFR) higher than 50 mL/min/1.73m^2^, despite of 3 to 6 months of optimized SC.^14^ Because patients with IgAN with an eGFR less than 50 mL/min/1.73m^2^ had been excluded in most previous trials, there is no recommendation for such patients in current clinical practice.

Mycophenolate mofetil (MMF) is a potent immunosuppressive agent that is relatively selective for lymphocytes and inhibits antibody production by B cells more strongly than any other immunosuppressant.^15^ However, data regarding the efficacy of MMF treatment in patients with IgAN are controversial.^10,11,12^ This study was designed to test the hypothesis that the addition of MMF to comprehensive SC would be superior to SC alone in reducing the risk of clinically important kidney outcomes in patients with IgAN and at high risk of progressing to ESKD.

## Methods

The study protocol for this RCT was approved by the Nanfang Hospital Medical Ethics Committee, and all patients provided written informed consent. We report the study following the Consolidated Standards of Reporting Trials (CONSORT) reporting guideline for RCTs.

### Study Design

The Effect of Mycophenolate Mofetil on Renal Outcomes in Advanced Immunoglobulin A Nephropathy (MAIN) was a prospective, open-label, RCT with a blinded end point conducted at the Renal Division of Nanfang Hospital, a center for kidney disease care in southern China. The principal investigator (F.F.H.) and steering committee designed the study protocol. An adjudicating committee, whose members were unaware of patient treatment assignments, adjudicated end point events.

A study period of 3 years was chosen on the basis of results of previous trials involving patients with nondiabetic chronic kidney disease (CKD), which suggested that a 3-year follow-up was adequate to assess efficacy.^16,17^ The detailed study protocol is provided in Supplement 1.

### Recruitment and Run-in Phase

Between September 2013 and December 2015, 525 patients were screened for participating in the trial. Inclusion criteria included (1) aged 18 to 70 years, (2) biopsy-proven IgAN, (3) urinary protein excretion rate (UPER) greater than 1 g/d, and (4) eGFR less than 60 mL/min/1.73m^2^ or persistent hypertension, defined as blood pressure greater than 140/90 mm Hg in 2 visits at least 1 day apart or need of an antihypertensive drug. Major exclusion criteria included secondary, familial, crescentic IgAN, presence of other CKD, any prior immunosuppressive therapy, and eGFR less than 30 mL/min/1.73m^2^.

A total of 238 qualified patients entered a 12-week run-in period. During this period, patients were treated with comprehensive SC, including blockade of RAS by use of losartan to reduce blood pressure to less than 130/80 mm Hg. In patients whose UPER remained at 0.75 g/d or greater despite blood pressure control, the dose of losartan was increased to the tolerable maximum daily dose as described previously.^17^ Patients who continued to show inadequate BP control were given an additional antihypertensive agent, excluding an RAS blocker. Patients with anemia and a hemoglobin level less than 11 g/dL were treated with erythropoietin (to convert to g/L, multiply by 10.0). Patients were also advised to undertake lifestyle modification, including quitting smoking, restricting salt intake (sodium chloride intake <5g/d), and avoiding nephrotoxic drugs. A statin was used when necessary. Dietary sodium intake was monitored by urinary sodium excretion rate (or chloride excretion rate in patients treated with sodium bicarbonate).

### Randomized Trial Phase

After the run-in phase, patients who had persistent proteinuria (UPER between 0.75 and 3.5 g/d) and who tolerated an RAS blockade (eGFR decline <30% greater than the baseline and without hyperkalemia) were randomly assigned at a 1:1 ratio to receive MMF plus SC (MMF group) or SC alone (SC group). Randomization was carried out via phone call.

Patients in the MMF group received orally administrated MMF at a daily dose of 1.5 g for 12 months and then tapered to a maintenance daily dose of 0.75 g to 1.0 g for at least 6 months. Both groups stayed on comprehensive SC as in the run-in period. During the trial phase, participants were followed up regularly every 2 to 3 months for a total of 3 years.

### Posttrial Phase

The trial cohort continued to be followed up regularly after the trial. In the posttrial phase, patients who did not initiate kidney replacement therapy continued to receive treatment with losartan and other SC. Patients in MMF group terminated MMF or were treated with a maintenance dose of MMF based on physician judgements or patient willingness.

### Laboratory Measurements

Serum creatinine levels were measured using an enzymatic method on an autoanalyzer (AU480; Beckman Coulter). UPER was determined by the Biuret method^18^ using 24-hour urine samples. We calculated eGFR with the creatinine-based CKD-EPI equation.^19^

### Study Outcomes

There were 2 primary outcomes: the time to a composite outcome and time to CKD progression. The composite outcome included doubling of serum creatinine level over the baseline level that was verified by another measurement at least 4 weeks apart; onset of ESKD defined by the need for maintenance dialysis or kidney transplant or kidney failure defined as an eGFR less than 15 mL/ min/1.73m^2^ and clinical indication for kidney replacement therapy; or death due to a kidney or cardiovascular cause. Progression of CKD was defined by a decrease in eGFR of 30% or more of the baseline and to a level of less than 60 mL/min/1.73m^2^ if the baseline eGFR was 60 mL/min/1.73m^2^ or greater or a decrease in eGFR of 50% or more of the baseline if the baseline eGFR was less than 60 mL/min/1.73m^2^.^20^ CKD progression was also verified by another measurement at least 4 weeks apart.

Secondary outcomes included (1) time to a 30% reduction in eGFR; (2) annual rate of eGFR loss; (3) the proportion of rapid kidney function decline, defined as a rate of eGFR decline of more than 5 mL/min/1.73m^2^/y; and (4) percentage change in UPER. Major outcomes measured in the posttrial phase were the annual rate of eGFR loss and changes in UPER.

### Statistical Analysis

Efficacy and safety analyses were performed using the intention-to-treat population. Baseline values of laboratory measurements were calculated as the mean of 2 values from the randomization visit and preceding visit.

#### Analyses of Primary Outcomes

The cumulative survival rates of 2 primary outcomes stratified by treatment group were estimated using the Kaplan-Meier method, and the group difference was tested by the log-rank test. Treatment effects on the 2 primary outcomes were estimated using Cox proportional hazard models, with and without covariate adjustment, respectively. Covariates included age, sex, body mass index (calculated as weight in kilograms divided by height in meters squared), systolic blood pressure, eGFR, UPER, and individual Oxford mesangial and endocapillary hypercellularity, segmental sclerosis, and interstitial fibrosis/tubular atrophy, and crescents (MEST-C) scores at baseline. Treatment effects were also estimated in subgroups stratified by sex, age, presence of hypertension, individual MEST-C scores, and eGFR and UPER at baseline. Modification of the treatment effect by the stratifying factor was tested using an analysis of variance test.

#### Analyses of Secondary Outcomes

The difference in the percentage change in the UPER during the trial period between treatment groups was tested using a 2-sample *t* test. The treatment effect on eGFR slope (annual absolute change in eGFR) was estimated using a linear mixed effect model. The treatment effect on a 30% reduction in eGFR was estimated using a Cox proportional hazard model with and without covariate adjustment. The treatment effect on rapid kidney function decline (>5 mL/min/1.73m^2^/y) was estimated using a logistic regression model with and without covariate adjustment.

#### Safety Analysis

Serious adverse events were summarized as the total count of events and the count (with percentage) of patients experiencing at least 1 event. Differences in the proportions of patients experiencing at least 1 event were tested using Fisher exact test.

#### Posttrial Analysis

Mean eGFR slopes during the entire study period (trial and after trial) in MMF and SC groups were estimated and compared using a linear mixed effect model. Mean eGFR slopes during the posttrial period were also estimated and compared among participants in the SC group, participants who discontinued MMF during the follow-up period, and those who continued MMF treatment throughout the follow-up period.

For the primary analysis, a 2-sided *P* value < .025 was regarded as statistically significant in order to correct for multiple testing. The 95% CIs for effect sizes associated with primary outcomes were not adjusted for multiple testing. For secondary and safety analyses, multiple testing adjustment was not applied, and a 2-sided *P* value < .05 was regarded as statistically significant. Analyses were performed using R statistical software version 4.1.2 (R Project for Statistical Computing). Data were analyzed from March through June 2022.

## Results

**Figure 1.**
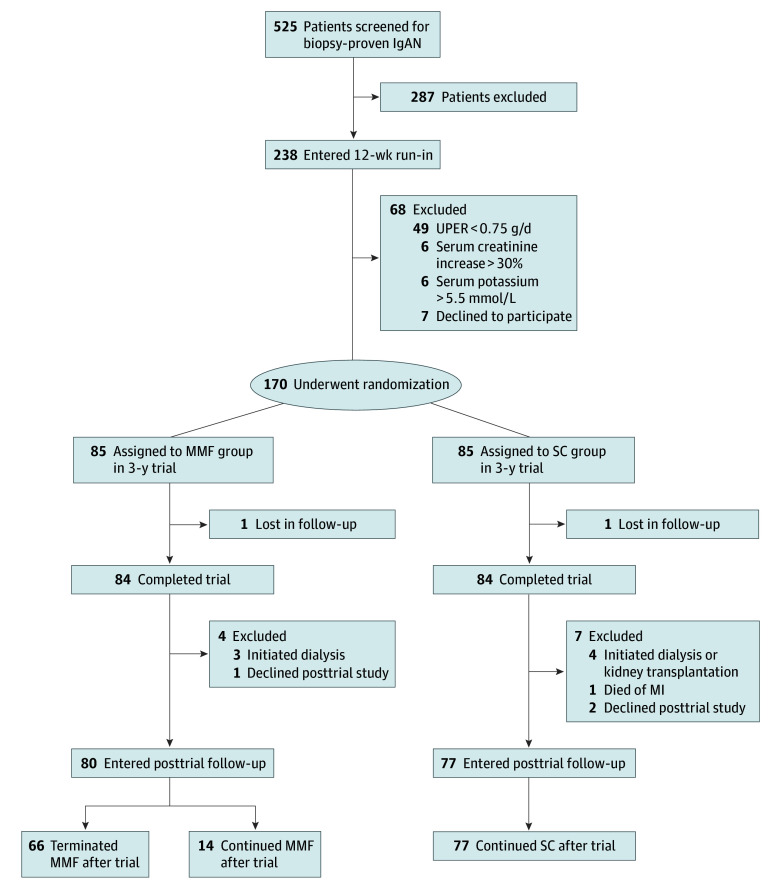
Study Flowchart IgAN indicates immunoglobulin A nephropathy; MI, myocardial infarction; MMF, mycophenolate mofetil; SC, supportive care; UPER, urinary protein excretion rate.

A total of 238 patients entered the 12-week run-in period, among whom 49 patients had an UPER that decreased to less than 0.75 g/d after SC, 12 patients did not tolerate losartan, and 7 patients declined to undergo randomization (Figure 1). Of the remaining 170 participants (mean [SD] age 36.6 [9.4] years; 94 [55.3%] male patients), 85 patients were randomly assigned to receive MMF with SC (MMF group) and 85 patients to receive SC alone; 168 patients (98.8%) completed the trial. Both groups were treated with optimized SC as in the run-in period for 3 years. Patients in the MMF group additionally received MMF at a daily dose of 1.5 g for 12 months, followed by a maintenance daily dose of 0.75 to 1.0 g for at least 6 months. The mean (SD) dose and treatment duration of MMF were 1.0 (0.2) g and 30 (18) months, respectively, with a mean (SD) compliance rate of 94.3% (12.4%). Characteristics of participants at randomization are summarized in Table 1. The mean (SD) eGFR was 50.1 (17.9) mL/min/1.73m^2^, and the mean (SD) proteinuria level was 1.9 (1.7) g/d. There were 153 participants (90.0%) received a diagnostic kidney biopsy within 1 year prior to randomization.

**Table 1.** Characteristics of Participants at Randomization

Characteristic	Participants, No. (%)		
	Total (N = 170)	MMF (n = 85)	SC (n = 85)
Age, mean (SD), y	36.6 (9.4)	35.0 (8.7)	38.2 (9.8)
Sex			
Males	94 (55.3)	42 (49.4)	52 (61.2)
Females	76 (44.7)	43 (50.6)	33 (38.8)
Hypertension	96 (56.5)	48 (56.5)	48 (56.5)
BMI, mean (SD)	23.1 (3.4)	23.3 (3.1)	22.9 (3.7)
Blood pressure, mean (SD), mm Hg			
Systolic	126.2 (18.3)	126.3 (17.8)	126.1 (18.8)
Diastolic	81.8 (13.0)	82.6 (13.7)	80.9 (12.4)
eGFR, mean (SD), mL/min/1.73 m^2^			
mean (SD)	50.1 (17.9)	50.9 (18.2)	49.3 (17.7)
category			
≥50	74 (43.5)	37 (43.5)	37 (43.5)
<50	96 (56.5)	48 (56.5)	48 (56.5)
Serum level, mean (SD)			
Albumin, g/dL	3.9 (0.5)	3.8 (0.6)	4.0 (0.5)
Triglycerides, mg/dL	165.5 (116.9)	165.5 (116.9)	165.5 (124.0)
Cholesterol, mg/dL	188.6 (49.2)	185.6 (49.2)	188.6 (49.2)
Urinary protein excretion, mean (SD), g/d	1.9 (1.7)	2.1 (1.9)	1.7 (1.3)
Microscopic hematuria	170 (100)	85 (100)	85 (100)
Pathologic finding			
GS ≥50%	106 (62.4)	53 (62.4)	53 (62.4)
Oxford classification^a^			
M1	170 (100)	85 (100)	85 (100)
E1	15 (8.8)	10 (11.8)	5 (5.9)
S1	142 (83.5)	75 (88.2)	67 (78.8)
T1	69 (40.6)	33 (38.8)	36 (42.4)
T2	92 (54.1)	46 (54.1)	46 (54.1)
C1	69 (40.6)	36 (42.4)	33 (38.8)
C2	3 (1.8)	2 (2.4)	1 (1.2)
Statin treatment	151 (88.8)	70 (82.4)	68 (80.0)
Diuretic	88 (51.8)	42 (49.4)	46 (54.1)
Daily dose of losartan in run-in period, mean (SD), mg	124 (48)	115 (39)	128 (54)

Abbreviations: BMI, body mass index (calculated as weight in kilograms divided by height in meters squared); eGFR, estimated glomerular filtration rate; GS, glomerular sclerosis; MMF, mycophenolate mofetil; SC, supportive care.

SI conversion factors: To convert albumin to grams per liter, multiply by 10.0; cholesterol to mmol per liter, multiply by 0.0259; triglycerides to millimoles per liter, multiply by 0.0113.

^a^ Kidney histologic lesions were graded according to Oxford classification.

The mean (SD) daily dose of losartan was comparable between the MMF and the SC group at randomization (Table 1) and during trial phase (119 [40] mg vs 127 [55] mg). During the 3-year trial phase, 2 participants were lost to follow-up, 1 each from the MMF and SC groups.

### Outcomes in Trial Phase

#### Primary Outcomes

**Figure 2.**
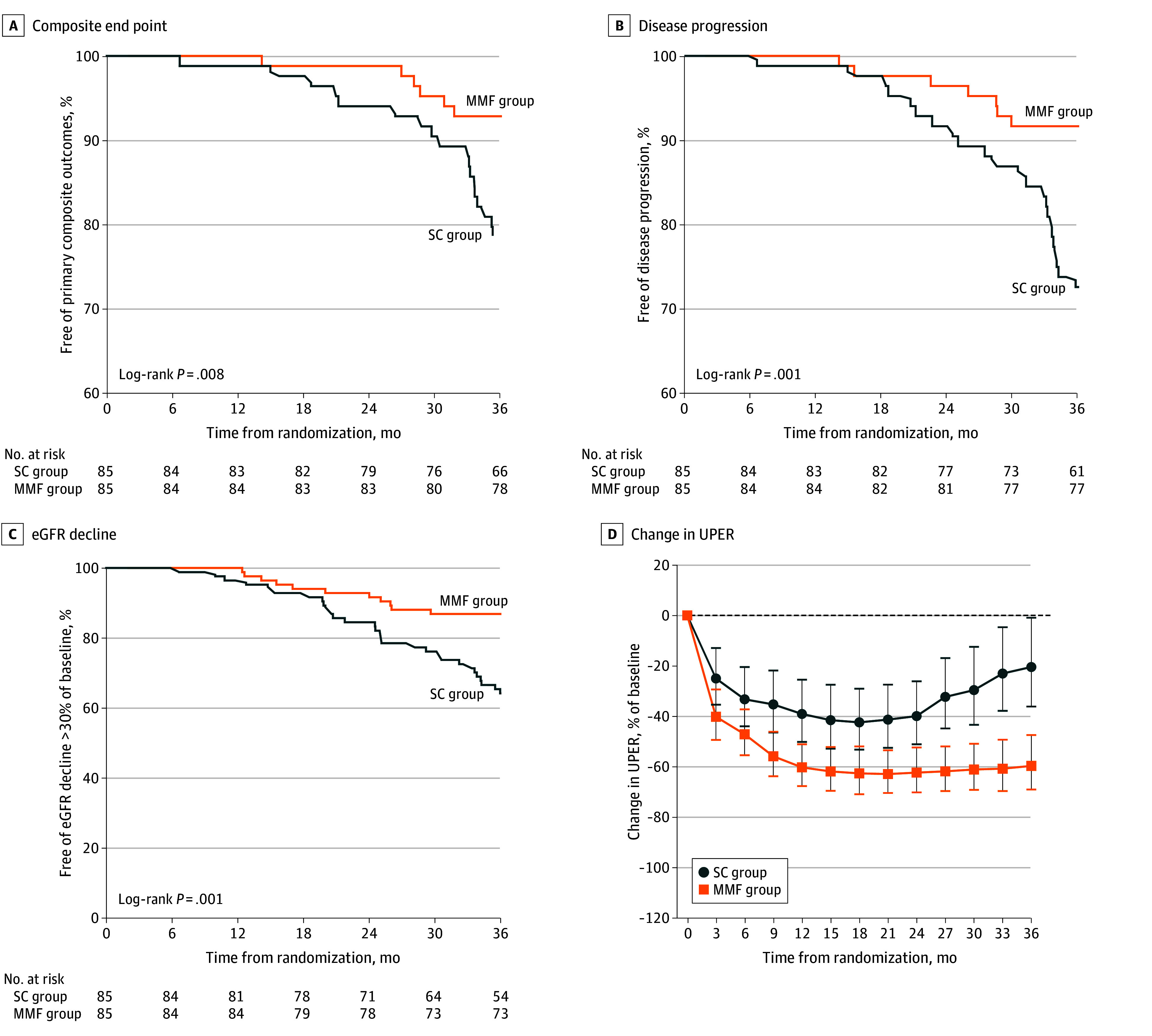
Primary and Secondary Outcomes A, Primary composite outcomes of Kaplan-Meier estimates of the percentage of patients free of the primary composite end point of a doubling of serum creatinine levels, end-stage kidney disease, or death due to kidney or cardiovascular cause are presented. B, Primary outcomes of Kaplan-Meier estimates of the percentage of patients without disease progression are presented. C, Secondary outcomes of Kaplan-Meier estimates of the percentage of patients without an estimated glomerular filtration rate (eGFR) decline of more than 30% of baseline are presented. D, Secondary outcomes of a change in urinary protein excretion rate (UPER; percentage of baseline) are presented. Horizontal line indicates reduction in proteinuria from baseline; MMF, mycophenolate mofetil; SC, supportive care.

**Figure 3.**
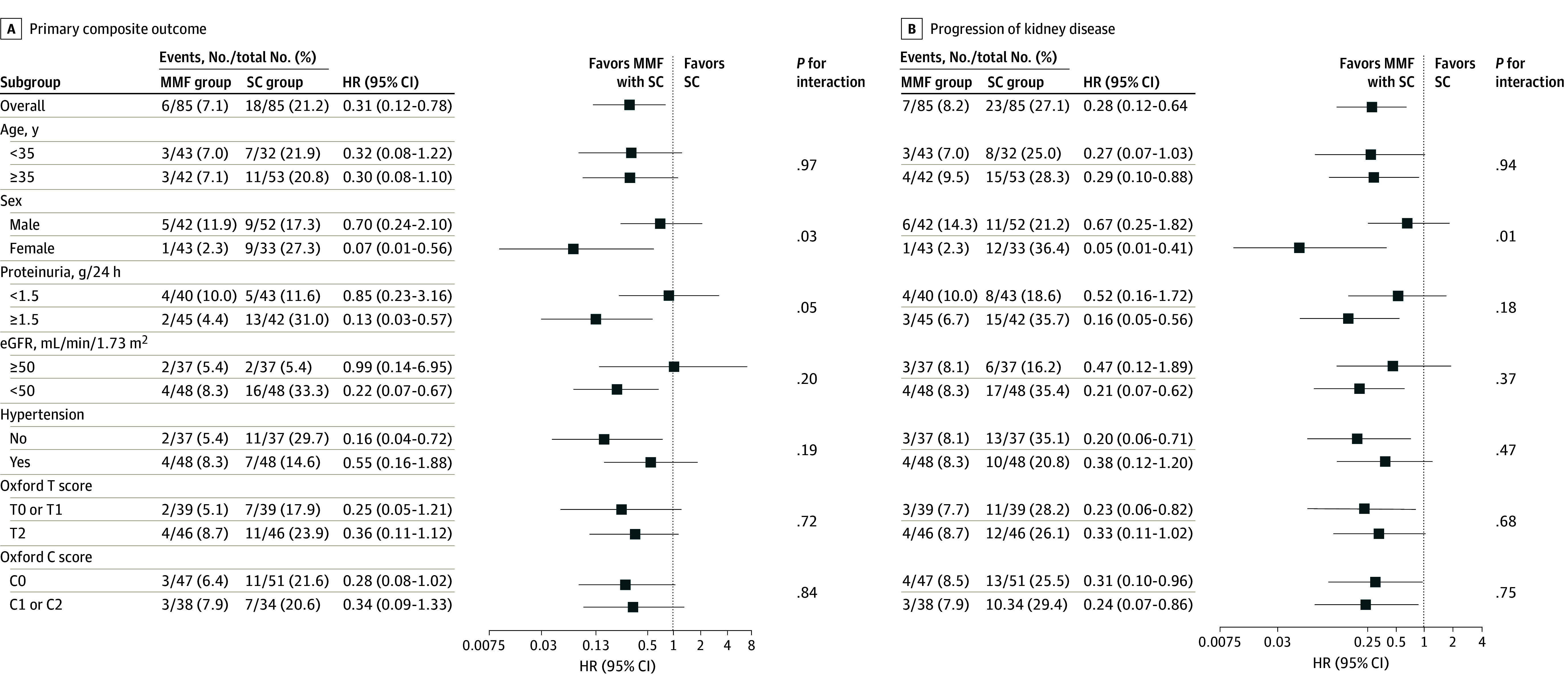
Primary Outcomes by Prespecified Subgroup at Baseline A, The forest plot of hazard ratios (HRs) for the primary composite outcome are shown. B, The forest plot of HRs for progression of chronic kidney disease are shown. eGFR indicates estimated glomerular filtration rate; MMF, mycophenolate mofetil; SC, supportive care.

The primary composite outcomes were observed in 24 participants, including 6 patients in the MMF group (7.1%) and 18 patients in the SC group (21.2%; log-rank *P* = .008) (Figure 2A). There was 1 death in the SC group due to myocardial infarction. Similarly, CKD progression occurred in 7 participants in the MMF group (8.2%) and 23 participants in the SC group (27.1%; log-rank *P* = .001) (Figure 2B). MMF treatment reduced risk of the composite outcome and disease progression by 77% (adjusted hazard ratio [aHR], 0.23; 95% CI, 0.09-0.63) and 77% (aHR, 0.23; 95% CI, 0.10-0.57), respectively (Table 2). Treatment effects were generally consistent across prespecified subgroups (Figure 3).

**Table 2.** Components of Primary Outcomes

Outcome	Participants, No. (%) (N = 170)		Unadjusted HR (95% CI)	*P* value	Adjusted HR (95% CI)^a^	*P* value
	MMF (n = 85)	SC (n = 85)				
Primary composite outcome^a^	6 (7.1)	18 (21.2)	0.31 (0.12-0.78)	.01	0.23 (0.09-0.63)	.004
Doubling of serum creatinine	6 (7.1)	18 (21.2)	0.31 (0.12-0.78)	.01	0.23 (0.09-0.63)	.004
ESKD	3 (3.5)	7 (8.2)	0.41 (0.11-1.58)	.20	0.27 (0.06-1.21)	.09
Kidney failure	0 (0.0)	3 (3.5)	NA	NA	NA	NA
Maintained dialysis	3 (3.5)	3 (3.5)	NA	NA	NA	NA
Kidney transplant	0 (0)	1 (1.2)	NA	NA	NA	NA
Death due to cardiovascular cause	0 (0)	1 (1.2)	NA	NA	NA	NA
CKD progression	7 (8.2)	23 (27.1)	0.28 (0.12-0.65)	0.003	0.23 (0.10-0.57)	.001

Abbreviations: CKD, chronic kidney disease; ESKD, end-stage kidney disease; HR, hazard ratio; MMF, mycophenolate mofetil; NA, not applicable; SC, supportive care.

^a^ Adjusted by age, sex, body mass index (calculated as weight in kilograms divided by height in meters squared), systolic blood pressure, baseline estimated glomerular filtration rate, urinary protein excretion rate, and Oxford mesangial and endocapillary hypercellularity, segmental sclerosis, and interstitial fibrosis/tubular atrophy, and crescents (MEST-C) score.

#### Secondary Outcomes

The percentage of patients without an eGFR decline greater than 30% of baseline in MMF and SC groups is shown in Figure 2C. There were 11 patients (12.9%) in the MMF group and 30 patients (35.3%) in the SC group with a 30% or greater reduction in eGFR (eTable 1 in Supplement 2). MMF treatment reduced the risk of a 30% reduction in eGFR by 72% (aHR, 0.28; 95% CI, 0.13-0.58).

Compared with the SC group, the MMF group had a significantly lower mean (SD) annual rate of eGFR decline (1.2 [0.6] vs 3.8 [0.6] mL/min/1.73m^2^/y; *P* < .001). There were 12 patients (14.1%) and 28 patients (32.9%) with annual eGFR decline greater than 5mL/min/1.73m^2^ in the MMF and SC groups, respectively. MMF treatment reduced the risk of rapid decline in eGFR by 75% (adjusted odds ratio, 0.25; 95% CI, 0.11-0.57).

The MMF group had a significantly higher rate of reduction in UPER from baseline compared with the SC group (1.2 g/d/2.1 g/d [57.1%] vs 0.5 g/d/1.7 g/d [28.2%[; *P* < .001). The changes in UPER during the trial phase stratified by treatment group are presented in Figure 2D.

### Adverse Events

The total counts of serious adverse events and all adverse events were comparable in the 2 treatment groups (eTable 2 in Supplement 2). Gastrointestinal symptoms, such as abdominal distension and diarrhea, were more common in the MMF group, particularly at early stages of treatment, but were tolerable in most patients. Infection events, predominantly pneumonia, were more frequently observed in the MMF group, although the difference between groups was not statistically significant. There were 14 patients (16.5%) with infections in the MMF group and 9 patients (10.6%) in the SC group. No fatal adverse event occurred in this trial.

### Outcomes in Posttrial Phase

A total of 157 participants (92.4%; 80 participants in the MMF group and 77 participants in the SC group) who had completed the trial and did not initiate kidney replacement therapy were followed up for a median (IQR) length of 60 (47-76) months. In the MMF group, 14 participants continued MMF treatment throughout the entire follow-up period, while 66 participants discontinued MMF after the trial phase.

In the study period (trial and after trial), the mean (SD) annual loss of eGFR was 2.9 (1.0) and 4.6 (0.9) mL/min/1.73m^2^ in the MMF and SC groups, respectively (*P* < .001). During the posttrial period, the mean (SD) annual loss of eGFR was 7.1 (1.0) mL/min/1.73m^2^ among 77 patients in the SC group, 6.1 (1.2) mL/min/1.73m^2^ among 66 patients who were randomized to MMF during the trial but discontinued MMF during the posttrial phase, and 4.1 (0.8) mL/min/1.73m^2^ among 14 patients who were randomized to MMF during the trial and continued MMF treatment throughout the follow-up period. The mean eGFR value at randomization and the end of trial and posttrial phases, as well as the mean decrease in UPER stratified by treatment group, are presented in the eFigure in Supplement 2.

## Discussion

In this RCT of patients with IgAN who were at high risk of disease progression, the addition of MMF to SC significantly reduced the risk of the primary composite outcome of doubling of serum creatinine, ESKD requiring dialysis or kidney transplantation, or death due to kidney or cardiovascular cause compared with receiving SC alone. The subsequent 5-year follow-up in the MAIN cohort suggested that withdrawal of MMF may have accelerated eGFR loss, which may add further support for the conclusion.

The role of immunosuppression in the management of progressive IgAN remains highly controversial. It is difficult to compare results among previous trials examining immunosuppression in IgAN given substantial differences in baseline demography, treatment protocols, follow-up duration, and clinical end points.^7,8,9,10,11,12^ To our knowledge, the only 2 trials lasting as long as ours and with a comparable or larger sample size were the TESTING study^7,13^ and STOP-IgAN trial.^8^ Our results are consistent with those of the TESTING study, which found that corticosteroid therapy was associated with a lower risk of composite kidney outcomes (40% reduction in eGFR or ESKD or death due to kidney disease).^7,13^ These findings, however, contrast with those from the STOP-IgAN trial, which did not find benefits for kidney outcomes in the original RCT^8^ or during long-term follow-up.^21^ Similarly, data regarding the efficacy of MMF treatment in patients with IgAN are controversial, likely due to the small sample size in most published studies, differences in patient characteristics (eg, low or high risk of disease progression and with or without consistent blockade of RAS), and differences in study populations.^10,11,12,22^

A feature of the MAIN trial was that participants were at high risk of disease progression. The mean eGFR of participants in MAIN at the time of randomization (50.1 mL/per/min/1.73m^2^) was lower than those in the STOP-IgAN (59 mL/min/1.73m^2^) and TESTING (61.5 mL/min/1.73m^2^) trials, as well as many previous trials on immunosuppression in IgAN.^9,11,12^ For patients with IgAN who are at risk of disease progression, typically those with proteinuria levels greater than 0.75 to 1.0 g/d despite SC for 3 to 6 months, KDIGO guidelines suggest a 6-month full-dose corticosteroid treatment in patients with an eGFR greater than 50 mL/min/1.73m^2^, although there is a low level of evidence supporting this.^23^ Because patients with IgAN and an eGFR less than 50 mL/min/1.73m^2^ were excluded in most trials, KDIGO guidelines do not provided any recommendations for treating such patients. Our results may provide new evidence to bridge the gap, suggesting no recommendation for patients with IgAN and CKD (ie, eGFR <60 mL/min/1.73m^2^) in current clinical practice. One may argue that immunosuppression may become less effective when sclerotic and fibrotic changes have been evident. However, even in the presence of CKD, immunological factors could still be active and responsible for disease progression.^24^ Consistent with that notion, our subgroup analyses suggested that patients with an eGFR of 30 to 50 mL/min/1.73m^2^ benefited equally or more from MMF treatment compared with those with an eGFR of 50 mL/min/1.73m^2^ or more. Furthermore, results from a large European cohort study^25^ suggested that corticosteroid therapy may be associated with kidney benefits for patients with IgAN with a baseline eGFR of less than 50 mL/min/1.73m^2^. Some reported data suggest a favorable outcome associated with immunosuppressive agents added to steroids.^26,27^

Another strength of the MAIN study was that large proportion (92.4%) of the trial cohort was followed up for a median of 60 months, which provided further information regarding the treatment of MMF in progressive IgAN. In the MAIN trial, the mean annual rate of decline in eGFR was 1.2 mL/min/1.73m^2^ in the MMF group compared with 3.8 mL/min/1.73m^2^ in the SC group (*P* < .001). However, in the posttrial phase, the annual rate of decline in eGFR in patients terminating MMF accelerated to 6.1 mL/min/1.73m^2^. This finding further suggests that the kidney benefit observed in the MMF group was attributable to MMF treatment and that the beneficial effect of MMF may not last long after drug withdrawal. This may explain why in a STOP-IgAN follow-up study,^21^ no difference was found between immunosuppression and SC groups, given that it was a purely observational study and no systemic therapy was administered. Consistently, data from the STOP-IgAN follow-up study did not show a legacy effect after a 6-month course of corticosteroids or a 3-year course of combined immunosuppression.^21^

In our 8-year study, a mean of 30 months of treatment with low-dose MMF (mean dose, 1.0 g/d) in addition to SC offered more kidney benefits than SC alone and was generally well tolerated. Immunosuppression has long been known to increase the risk of infections, as was observed in the STOP-IgAN study^8^ and the original full-dose corticosteroid group in the TESTING study.^7^ The risk of infection could be reduced by lowering the dose of immunosuppressive agents.^13^ The infection rate in our patients receiving MMF at an mean daily dose of 1 g was 16.5%, which was lower than the rates of 54% and 79% observed in trials using 2 or 3 g, respectively of MMF per day.^28,29^ Although there was no statistically significant difference between MMF and SC groups in the total number of adverse events, infection events, particularly pneumonia, were more frequent in the MMF group compared with the SC group. This needs to be highlighted, especially given that clinicians may be tempted to use MMF at more typical doses of 2g/d or higher. Consistent with our findings, long-term treatment with a reduced dose of MMF has been shown to be the most effective strategy to maintain remission in proliferative lupus nephritis^30^ and was used to prevent acute rejection in recipients of kidney transplantations.^31,32^

### Limitations

This study has several limitations. First, the study was an open-label trial. However, end points used were based on laboratory measurements and adjudicated by investigators who were blind to the treatment assignment. Furthermore, the rate of loss to follow-up was low and treatment compliance was high and well balanced between treatment groups; this suggests that the risk of investigator and participant bias would be small. Second, patients with proteinuria levels greater than 3.5 g/d were excluded from the study; therefore, we did not evaluate the efficacy of the treatment regimen in this population. Third, a single-center study may increase the risk of benefit overestimation. However, the consistency among multiple outcomes and analyses increases the robustness of our findings. Fourth, the study was conducted among patients in China, and so caution should be used when generalizing findings to other populations. However, the prespecified subgroup in the TESTING trial^13^ showed that the benefits of immunosuppression were comparable in participants who were not Chinese.

## Conclusions

Among patients with IgAN who were at high risk of progression, this RCT found that the addition of MMF to SC significantly reduced the risk of the primary composite outcome of doubling of serum creatinine, ESKD, or death due to kidney or cardiovascular caused compared with SC alone. The kidney protective effect of MMF was similar across prespecified subgroups. These results suggest that the addition of MMF to optimized SC was superior to SC alone in improving kidney outcomes and may be an alternative therapy for patients with IgAN, particularly those with CKD and subnephrotic proteinuria despite receiving SC, as well as those not appropriate for steroid therapy.

## Notes

Supplement 1.
Trial Protocol and Statistical Analysis Plan

Supplement 2. **eTable 1.** Secondary Outcomes **eTable 2.** Summary of Major Adverse Events **eFigure.** Mean Estimated Glomerular Filtration Rate and Changes in Urinary Protein Excretion in Trial and Posttrial Phases

Supplement 3.
Group Authors

Supplement 4.
Data Sharing Statement
