# Supplementary material for: Effectiveness of Mycophenolate Mofetil Among Patients With Progressive IgA Nephropathy: A Randomized Clinical Trial
Source: JAMA Netw Open. 2023 Feb 6;6(2):e2254054. doi: 10.1001/jamanetworkopen.2022.54054 (PMC12578496; doi:10.1001/jamanetworkopen.2022.54054)
Supplement: Supplement 1. — Trial Protocol and Statistical Analysis Plan [file jamanetwopen-e2254054-s001.pdf]

Clinical Trial Protocol Title Page

**Acronym/Title:**	**MAIN** / The Effect of **M**ycophenolate Mofetil on Renal Outcomes in **A**dvanced **I**mmunoglobulin A **N**ephropathy
**Protocol version and date:**	V2.0, 15 Nov 2017
**Clinicaltrials.gov study number:**	NCT01854814
**Study type / Study phase:**	Randomized Controlled Trial / Phase IV
**Study Initiator and Funder:**	Renal Division of Nanfang Hospital, Southern Medical University
**Country of study:**	China
**Author:**	Fan Fan Hou, et. al

The study will be conducted in compliance with the protocol and any applicable regulatory requirements.

This document contains information that is privileged or confidential and may not be disclosed for any purposes without the prior written consent of Renal Division of Nanfang Hospital, Southern Medical University

1. Table of contents

1. Table of contents **2**
2. List of abbreviations **4**
3. General information **5**
4. Abstract **5**
5. Rationale and background **8**
6. Trial hypotheses and objectives **8**
7. Research methods **8**
7.1 Study design 8
7.2 Study drug 9
7.3 Concomitant medication 9
7.4 Sample size 10
7.5 Randomization 10
7.6 Blind 10
7.7 Study Outcomes 10
Primary outcomes 10
Secondary outcomes 10
Safety endpoints 11
7.8 Setting 11
Eligibility 11
Inclusion and exclusion criteria 11
Withdrawal or study discontinuation 12
7.9 Visits 12
7.10 Trial procedure 1
By visit 1
Physical examination & Vital signs 2
Height and weight 2
Laboratory evaluation 2
7.11 Data management 3
7.12 Data analysis 3
Statistical considerations 3
7.13 Quality control 3
Data quality 3
Quality review 4
Storage of records and archiving 4
8 Protection of human subjects **4**
Ethical conduct of the study 4
Independent ethics committee (IEC) or institutional review board (IRB) 4
Patient information and consent 5
Confidentiality 5
9 Management and reporting of adverse events/adverse reactions **5**
Definitions 5
Collection 7
Management and reporting 7
Evaluation 8

10 References 8

2. List of abbreviations

AE	Adverse Event
AR	Adverse Reaction
BMI	Body Mass Index
CRF	Case Report Form
DBP	Diastolic Blood Pressure
DMP	Data Management Plan
eGFR	Estimated Glomerular Filtration Rate
EDC	Electronic Data Capture
ESRD	End Stage Renal Disease
HPF	High Power Field
HREC	Human Research Ethics Committee
ID	Identification
IEC	Independent Ethics Committee
IgA	Immunoglobulin A
IRB	Institutional Review Board
ITTs	Intention-to-Test Set
KDIGO	Kidney Disease Improving Global Outcomes
MedDRA	Medical Dictionary for Regulatory Activities
MMF	Mycophenolate Mofetil
MRP	Medical Review Plan
PI	Principal Investigator
QRP	Quality Review Plan
RAS	Renin-Angiotensin System
RBCs	Red Blood Cells
SAE	Serious Adverse Event
SAP	Statistical Analysis Plan
SBP	Systolic Blood Pressure
SDV	Source Data Verification
SOP	Standard Operating Procedure
SS	Safety Set
UPE	Urine Protein Excretion
WBCs	White Blood Cells

3. General information

Sponsor & Contact details:

Nanfang Hospital, Southern Medical University

4. Abstract

**Acronym/Title**	**MAIN** / The Effect of **M**ycophenolate Mofetil on Renal Outcomes in **A**dvanced **I**mmunoglobulin A **N**ephropathy
**Research objectives**	The study is to evaluate the effect of adding Mycophenolate Mofetil (MMF) to comprehensive supportive care, compared with supportive care alone, on a composite outcome of doubling of serum creatinine, end stage renal disease, and death due to kidney or cardiovascular cause, in patients with advanced IgA nephropathy.
**Study outcomes**	**Primary outcome:**<br>1. The time to the first event in the composite outcomes of doubling of serum creatinine, defined as two serum creatinine levels measured at four weeks apart that were twice the baseline value, the onset of end stage renal disease (ESRD), defined by the need for maintained dialysis or renal transplant or renal failure (defined as eGFR < 15 ml per min per $1.73m^2$ and need renal replacement therapy, but renal replacement therapy is not available or the patient refuse to receive renal replacement therapy), and death due to kidney or cardiovascular cause.<br><br>2. The time to progression of chronic kidney disease, defined by a decrease in eGFR of 30% or more from baseline and to a level of less than 60 ml per minute per $1.73m^2$ if the baseline eGFR was 60 ml per minute per $1.73m^2$ or more, or a decrease in eGFR of 50% or more if the baseline eGFR was less than 60 ml per minute per $1.73m^2$.<br><br>**Secondary outcomes:**<br>1. The time to 30% reduction in eGFR from the baseline.<br>2. Annual absolute changes in the eGFR (eGFR slope).<br>3. Rapid eGFR decline (<-5 ml/min/$1.73m^2$/year).<br>4. Percentage change in the rate of urinary protein excretion over the baseline.
**Population**	The target population will be patients with biopsy-proven IgA nephropathy and at high risk of progressing to ESRD.<br><br>**Inclusion criteria before run-in period:**<br>1. Adult female or male (18 years of age or older);<br>2. Biopsy-proven primary IgA nephropathy

	3. Urinary proteinuria excretion rate over 1g/24 hour<br>4. eGFR <60 ml/min/1.73m^2, or persistent hypertension, defined as blood pressure over 140/90 mmHg in two visits at least one day apart or need of antihypertensive drug<br>5. Signed informed consent<br><br>**Exclusion criteria before run-in period:**<br>1. Familial IgA nephropathy<br>2. Concomitant disease: cancer, active infection, including HBV infection (HBsAg-positive or HBeAg-positive, or serum detectable HBV-DNA) or clinical evidence active tuberculosis (nodules, cavities, tuberculoma, etc.), diabetes mellitus, connective tissue disease, liver disease, abnormal liver function<br>3. eGFR<30 ml/min/1.73m^2<br>4. Severe hypertension (systolic blood pressure over 180 mmHg and/or diastolic blood pressure over 110 mmHg)<br>5. Serum albumin level < 30g/L<br>6. Hyperkalemia (blood potassium ≥ 5.5 mmol/l)<br>7. Prior immunosuppressive therapy<br>8. Inability to comply with study and follow-up procedures<br>9. Pregnant women<br><br>**Inclusion criteria for randomization:**<br>Urinary protein excretion rate remained above 0.75g per day and lower than 3.5g per day, despite of 3 months treatment with supportive care<br><br>**Exclusion criteria for randomization:**<br>Not tolerable to RAS blockade (eGFR decline over 30% of baseline or developing hyperkalemia) during run-in phase; eGFR lower than 30 ml/min/1.73m^2 after run-in period
**Study design**	This study is a prospective, open-label, randomized controlled, blinded endpoint (PROBE) trial.<br>During a 12-week run-in period, all patients will be treated with comprehensive supportive care, including blockade of renin-angiotensin system (RAS) by using losartan to reduce blood pressure to a target below 130/80 mmHg. In patients whose urinary protein excretion rate remained above 0.75g per day despite blood pressure control, the dose of losartan will be increased to the tolerable maximum daily dose as described previously. Patients will be advised for lifestyle modification to quit smoking, restrict high-salt intake (sodium chloride intake < 5g per day), and avoid nephrotoxic drugs. Anemic patients with a hemoglobin level lower than 110g/L were treated with erythropoietin. Statin was used when necessary. Dietary sodium intake were monitored by urinary sodium excretion rate (or chloride excretion rate in patients treated with sodium bicarbonate) measured by 24-hour urine sample.<br>By the end of run-in period, patients who have persistent proteinuria, defined as urinary protein excretion rate above 0.75g

	and lower than 3.5g per day, and those who are willing to participate the study entered the 3-year trial phase and will be randomly assigned to MMF plus supportive care or to supportive care alone. Patients who are not tolerable to RAS blockade (eGFR decline over 30% of baseline or developing hyperkalemia) during run-in phase will not undergo randomization.<br>In the trial phase, patients assigned to the MMF group received oral administrated MMF at a daily dose of 1.5g for 12 months and then tapered to a maintained daily dose of 0.75g to 1.0g for at least 6 months. Both MMF plus supportive care and supportive care alone group stayed on supportive care as in run-in period.
**Study size**	The sample size is estimated before the study with the use of nQuery Advisor software. Our preliminary study of IgA nephropathy showed that the three-year rate of the primary endpoint (doubling of serum creatinine, end-stage renal disease) among patients with IgA nephropathy taking RAS inhibitors was 36%. It is presumed that MMF treatment will reduce this rate to 16%. Thus, the enrollment of 76 per group will provide the study with a statistical power of 80% at a two-sided significance level of 0.05. Considering 10% of participants lost to follow-up over 3 years, the final sample size was 84 per group.
**Efficacy assessments**	1. Composite outcomes of a doubling of serum creatinine, ESRD, or death due to kidney or cardiovascular cause<br>2. Time to progression of chronic kidney disease<br>3. The time to 30% reduction in eGFR from the baseline.<br>4. Annual absolute changes in the eGFR (eGFR slope) over the trial period.<br>5. Rapid eGFR decline (<-5 ml/min/1.73m^2/year).<br>6. Percentage change in the rate of urinary protein excretion at the end of trial over the baseline.
**Safety assessments**	All adverse events (AEs), serious adverse events (SAEs), study drug-related SAEs, AEs leading to study withdrawal, AEs leading to study withdrawal, and AEs related to the study drug

5. Rationale and background

Immunoglobulin A (IgA) nephropathy is the most common type of glomerulonephritis. Up to 40% of patients with IgA nephropathy progress to end stage renal disease (ESRD) over 10 to 20 years, making it the leading cause of kidney failure in many parts of the world.[1]

The appropriate therapy of IgA nephropathy remains uncertain. The central role of the immune and autoimmune activation in the pathogenesis of IgA nephropathy [2,3] indicates a potential benefit of immunosuppression for treating the disease. However, the efficacy of immunosuppression in IgA nephropathy have not yielded confirmed results.[1,4] Current management of IgA nephropathy remains focused on non-immunosuppressive-based strategies, so-called supportive care, to reduce proteinuria and slow the disease progression. This encompasses optimal inhibition of renin-angiotensin system (RAS), rigorous blood pressure control, and lifestyle modification.[4] However, despite intensive supportive care, considerable number of patients still have massive proteinuria and remain at high risk of disease progression. The Kidney Disease Improving Global Outcomes (KDIGO) guidelines suggest the use of systemic glucocorticoids in patients who have a proteinuria level above 1g per day and an estimated glomerular filtration rate (eGFR) higher than 50ml per minutes per 1.73m^2, despite of 3 to 6 months of optimized supportive care.[5] The benefits of immunosuppressive therapy, particularly in those with an eGFR < 50 ml/min/1.73m^2, remain largely unclear.

Mycophenolate mofetil (MMF) is a potent immunosuppressive agent which is relative selective for lymphocytes and inhibits antibody production by B cells stronger than any other immunosuppressants.[6] However, data regarding the efficacy of MMF treatment in patients with IgA nephropathy are controversial, probably due to the small sample size in most published studies, and differences in patients characteristics, e.g. low or high risk of disease progression, and with or without consistent blockade of RAS. [5, 7, 8]

6. Trial hypotheses and objectives

The present study is designed to test the hypothesis that addition of MMF to comprehensive supportive care would be superior to supportive care alone in reducing the risk of clinical important kidney outcomes in patients with IgA nephropathy and at high risk of progressing to ESRD.

7. Research methods

7.1 Study design

This study is a prospective, open-label, randomized controlled, blinded endpoint (PROBE) trial. This trial will include adult patients with IgA nephropathy who are at high risk of renal progression.

During a 12-week run-in period, all participants will be treated with comprehensive supportive care, including blockade of RAS using losartan to reduce blood pressure to a target below 130/80 mmHg. In participants whose urinary protein excretion rate remained above 0.75g per day despite blood pressure control, the dose of losartan will be increased to the tolerable maximum daily dose as described previously [9]. Participants will be advised for lifestyle modification to quit smoking, restrict high-salt intake, and avoid nephrotoxic drugs. Anemic patients with a hemoglobin level lower than 110g/L were treated with erythropoietin. Statin was used when necessary. Dietary sodium intake

were monitored by urinary sodium excretion rate (or chloride excretion rate in patients treated with sodium bicarbonate) measured by 24-hour urine sample.

By the end of run-in period, participants who have persistent proteinuria, defined as urinary protein excretion rate above 0.75g and lower than 3.5g per day, eGFR over 30 ml/min/1.73m^2, and those who are willing to participate the study entered the 3-year trial phase and will be randomly assigned to MMF plus supportive care or to supportive care alone. Participants who are not tolerable to losartan (eGFR decline over 30% of baseline or developing hyperkalemia) during run-in phase will not undergo randomization.

Participants will be randomly assigned to the MMF plus supportive care group received oral administrated MMF at a daily dose of 1.5g for 12 months and then tapered to a maintained daily dose of 0.75g to 1.0g for at least 6 months. Both MMF plus supportive care and supportive care alone group stayed on supportive care as in run-in period. Participants will continue to be followed at regular intervals for a planned 3 years.

7.2 Study drug

(1) Drug information

- Drug: Mycophenolate mofetil (MMF)
- Formulation: Mycophenolate mofetil tablets 0.25g/tablet
- Manufacturer: Hangzhou Zhongmei Huadong Pharmaceutical Co., Ltd

(2) Drug accountability

The trained delegated study staff will acknowledge receipt of all shipments of the study treatments by emailing the signed investigator product receipt form contained in the shipment to the study center. The study treatments must be kept in a locked area with restricted access. The study treatments must be stored and handled in accordance with the manufacturer's instructions. The investigator or pharmacist will also keep accurate records of the quantities of the study treatments dispensed, used, and returned by each participant using an accountability form.

(3) Participant compliance

Study medications will be distributed by the investigator or appropriately qualified designee. Participants will be instructed to bring their unused study drug to every visit. Compliance will be assessed by tablet counts with regard to the total number of tablets taken over the entire treatment period. Investigators and their study personnel will be instructed to be sure that all participants take their prescribed number of tablets each month. If a participant forgets to take the tablets on a particular day she/he should be instructed to continue as planned on the next day. The participant should not try to catch up by increasing the dose on the next day.

7.3 Concomitant medication

Participants in this study, whether in the MMF or control arm, will all receive supportive care for IgA nephropathy. The investigator should strive to control the blood pressure to a target of 130/80 mmHg. Throughout the trial, all participants should receive losartan adjusted to the maximal tolerated dose. All participants will have dietary recommendations for CKD, e.g. Low-salt intake (<5 g per day). Participants will be advised to quit smoking and limit alcohol intake during the study as part of standard supportive care. Anemic patients with a hemoglobin level lower than 110g/L were treated with erythropoietin. Statins will be recommended when necessary. Dietary sodium intake

were monitored by urinary sodium excretion rate (or chloride excretion rate in patients treated with sodium bicarbonate) measured by 24-hour urine sample.

7.4 Sample size

The sample size is estimated before the study with the use of nQuery Advisor software. Our preliminary study of IgA nephropathy showed that the three-year rate of the primary endpoint (doubling of serum creatinine, end-stage renal disease, and death) among patients with IgA nephropathy taking RAS inhibitors was 36%. It is estimated that MMF treatment will reduce this rate to 16%. Thus, the enrollment of 76 per group will provide the study with a statistical power of 80% at a two-sided significance level of 0.05. Considering 10% of participants lost to follow-up over 3 years, the final sample size was 84 per group.

7.5 Randomization

All participants meeting inclusion and exclusion criteria and providing informed consent will be randomized to either the MMF plus supportive care group or supportive care alone group in a 1:1 ratio. A random sequence with a block size of 6 will be generated before the start of the trial and maintained by a party not involved in the conduct of the study. Randomization will be carried out via phone call.

7.6 Blind

This is an open-label, blinded end point trial. Both the participant and study personnel will be aware of the treatment assignment. An adjudicating committee, whose members will be unaware of patients' treatment assignments, review the data to determine which patient has reached study end points.

7.7 Study Outcomes

Primary outcomes

1. The time to the first event in the composite outcomes of doubling of serum creatinine, defined as two serum creatinine levels measured at four weeks apart that were twice the baseline value, the onset of end stage kidney disease (ESRD), defined as the need for maintenance dialysis or renal transplantation or renal failure (defined as eGFR < 15 ml per min per 1.73m^2 and need renal replacement therapy, but renal replacement therapy is not available or the patient refuse to receive renal replacement therapy), and death due to kidney or cardiovascular cause.

2. The time to progression of chronic kidney disease, defined by a decrease in eGFR of 30% or more from baseline and to a level of less than 60 ml per minute per 1.73m^2 if the baseline eGFR was 60 ml per minute per 1.73m^2 or more, or a decrease in eGFR of 50% or more if the baseline eGFR was less than 60 ml per minute per 1.73m^2. The chronic kidney disease progression endpoints were also verified by another measurement at least four weeks apart.

Secondary outcomes

1. Time to 30% reduction in eGFR from baseline
2. Annual rate of eGFR loss
3. Proportion of rapid decline in renal function defined as annual reduction rate of eGFR greater than 5 ml/min/1.73m^2/year.

4. Changes in the urinary protein excretion rate

Safety endpoints

1. Including all adverse events (AEs), serious adverse events (SAEs), study drug-related SAEs, AEs leading to study withdrawal, and AEs related to the study drug occurred during the study period will be recorded.

1. SAEs were defined as death, life-threatening conditions, requiring inpatient hospitalization or prolongation of existing hospitalization.

2. AEs with special concern were defined as drug-related AE listed in the drug information provided by the pharmaceutical manufacture of MMF, including leukopenia or leukocytosis, anemia, sepsis, gastrointestinal symptoms (diarrhea, stomachache, vomiting, anepithymia), certain types of repeated infections (pneumonia, influenza syndrome, urinary tract infection, herpes zoster, etc.), hepatic dysfunction or transaminase elevation, malignancy.

7.8 Setting

Eligibility

The target population will be patients with IgA nephropathy and at high risk of progressing to ESRD. Evidence of assessment of all eligibility criteria by the physician or a delegate, as well as enrollment of a patient in the study should be documented in the patient medical records.

Inclusion and exclusion criteria

Inclusion criteria before run-in period

1. Adult female or male (18 years of age or older);
2. Biopsy-proven primary IgA nephropathy
3. Urinary proteinuria excretion rate over 1g/24 hour
4. eGFR <60 ml/min/1.73m^2, or persistent hypertension, defined as blood pressure over 140/90 mmHg in two visits at least one day apart or need of antihypertensive drug
5. Signed informed consent

Exclusion criteria before run-in period

1. Familial IgA nephropathy
2. Concomitant disease: cancer, active infection, including HBV infection (HBsAg-positive or HBeAg-positive, or serum detectable HBV-DNA) or clinical evidence active tuberculosis (nodules, cavities, tuberculoma, etc.), diabetes mellitus, connective tissue disease, liver disease, abnormal liver function
3. eGFR<30 ml/min/1.73m^2
4. Severe hypertension (systolic blood pressure over 180 mmHg and/or diastolic blood pressure over 110 mmHg)
5. Serum albumin level < 30g/L
6. Hyperkalemia (blood potassium ≥ 5.5 mmol/l)
7. Prior immunosuppressive therapy
8. Inability to comply with study and follow-up procedures
9. Pregnant women

Inclusion criteria for randomization

Patients completed run-in, and with urinary protein excretion rate remaining above 0.75g per day but lower than 3.5g per day

Exclusion criteria for randomization

Patients not tolerable to losartan during run-in phase (eGFR decline over 30% of baseline or developing hyperkalemia), or eGFR < 30 ml/min/1.73m^2 after run-in

Withdrawal or study discontinuation

In this study, withdrawal from the study is independent of the underlying therapy and will not affect the patient's medical care. Each patient may withdraw from the study at any time and without giving a reason. The investigator also has the right to withdraw participants from the study treatment if they believe that is in the best interests of the participant due to intercurrent illness, SAE, treatment failure, protocol violations, non- compliance, administrative reasons or other reasons. If a patient wants to terminate the study participation, no further data will be collected. In case a patient would like to withdraw the consent given earlier, he/she should inform his/her doctor and the site should document the withdrawal in the (electronic) Case Report Form as well as in the patient medical records.

7.9 Visits

Information to be collected at the visits are summarized in Table 1.

Table 1: Tabulated overview on data collected during the study

Phase	Screening	Run-in				Treatment period																						
**Visits**	**1**	**2**	**3**	**4**	**5**	**6**	**7**	**8**	**9**	**10**	**11**	**12**	**13**	**14**	**15**	**16**	**17**	**18**	**19**	**20**	**21**	**22**	**23**	**24**	**25**	**26**	**27**	**28**
**Time (week)**	**-14**	**-12**	**-8**	**-4**	**0**	**4**	**8**	**12**	**16**	**20**	**28**	**36**	**44**	**52**	**60**	**68**	**76**	**78**	**86**	**94**	**102**	**110**	**118**	**126**	**134**	**142**	**150**	**156**
Informed consent form		×																										
In/exclusion criteria		×																										
Medical history/ demography	×																											
Height, weight	×	×	×	×	×	×	×	×	×	×	×	×	×	×	×	×	×	×	×	×	×	×	×	×	×	×	×	×
Physical examination	×	×			×																							×
Vital signs	×	×	×	×	×	×	×	×	×	×	×	×	×	×	×	×	×	×	×	×	×	×	×	×	×	×	×	×
Screening log	×																											
Randomization					×																							
Blood chemistry [1]	×		×		×	×	×	×	×	×	×	×	×	×	×	×	×	×	×	×	×	×	×	×	×	×	×	×
Hematology	×		×		×	×	×	×	×	×	×	×	×	×	×	×	×	×	×	×	×	×	×	×	×	×	×	×
Urinary analysis	×		×		×	×	×	×	×	×	×	×	×	×	×	×	×	×	×	×	×	×	×	×	×	×	×	×
24-hour urine protein [2]	×		×		×	×	×	×	×	×	×	×	×	×	×	×	×	×	×	×	×	×	×	×	×	×	×	×
Diet education	×	×	×	×	×	×	×	×	×	×	×	×	×	×	×	×	×	×	×	×	×	×	×	×	×	×	×	×
Study drug dispensation						×	×	×	×	×	×	×	×	×	×	×	×	×										
Concomitant medications	×	×	×	×	×	×	×	×	×	×	×	×	×	×	×	×	×	×	×	×	×	×	×	×	×	×	×	×
AEs/SAEs			×	×	×	×	×	×	×	×	×	×	×	×	×	×	×	×	×	×	×	×	×	×	×	×	×	×
Endpoints						×	×	×	×	×	×	×	×	×	×	×	×	×	×	×	×	×	×	×	×	×	×	×

1. Including: serum creatinine (CR), urea nitrogen (BUN), uric acid (UA), total carbon dioxide (tCO2), potassium (k), sodium (NA) and chlorine (CL), alanine aminotransferase (ALT), aspartate aminotransferase (AST), albumin (ALB), total bilirubin (T-Bil), triglyceride (TG), cholesterol (Chol), very low density lipoprotein (VLDL), high density lipoprotein (HDL), low density lipoprotein (LDL), calcium (CA), phosphorus (P), glucose (Glu). It is necessary to remind the patient at the first visit that blood should be taken on a fasting status at the next visit.

2. It is necessary to remind the patient to take a 24-hour urine sample at the next visit at the previous visit

Screening period

Visit 1 Days -14 to 1

All potentially eligible participants will be screened. The screening procedures to be performed are described in Table 1.

Run-in period

Visit 2-4

All eligible participants who provide informed consent will be invited to enter the run-in phase. The aim of 12- week run-in phase is to evaluate eligibility for the trial. Participants will be advised for lifestyle modification to quit smoking, restrict high-salt intake, and avoid nephrotoxic drugs. Statin was used when necessary.

Visit 5

If all inclusions are fulfilled on this visit, the participants are randomized. Patients will be randomly assigned to the MMF plus supportive care group in a 1:1 ratio.

Treatment period

Visit 6-18

Patients who are assigned to the MMF group will received oral administrated MMF at a daily dose of 1.5g for 12 months and then tapered to a maintained daily dose of 0.75g to 1.0g for at least 6 months. Patients who are assigned to the supportive care group will stay on supportive care as in run-in period. Participants will receive routine follow-up every 4 weeks in the first 6 months and then every 8 weeks.

Visit 19-28

During follow-up, participants will continue to receive routine follow-up every 8 weeks.

7.10 Trial procedure

By visit

Table 1 lists all of the assessments and indicates with an "X" the visits (data collection) when they are performed. Participants', who discontinue study drug before completing the study, should be encouraged to attend scheduled study visits for the duration of the follow-up. At a minimum, they will be contacted for safety evaluations during the 30 days following the last dose of study drug, including final contact at the 30-day point. Documentation of attempts to contact the patient will be recorded in the patient record.

All data obtained from the assessments listed in Table 1 must be supported in the patient's source documentation. Visit dates should be adhered to as closely as possible. If one visit is postponed or brought forward, it should not result in the next visit being postponed or brought forward. The next visit, if at all possible, should adhere to the original time schedule.

Physical examination & Vital signs

A complete physical examination will be performed at Visit 1 (table 1), and the last visit. It will include the examination of general appearance, skin, neck, eyes, ears, nose, throat, lungs, heart, abdomen, back, lymph nodes, extremities, vascular and neurological. A short physical exam will include the examination of general appearance and vital signs (BP, and pulse rate).

Height and weight

Height in centimeters (cm) will be measured at Visit 5 (randomization). Body weight (to the nearest 0.1 kilogram [kg] in indoor clothing, but without shoes) will be measured at every visit as listed in table 1.

Laboratory evaluation

Laboratory evaluation of all specimens will be performed according to visit procedure.

Renal endpoints that need determined by serum creatinine including doubling in serum creatinine, kidney failure have to be confirmed by two measurements at least 4-weeks apart. For this purpose, patients may need to attend an unscheduled visit one month after the study visit.

Laboratory values that exceed the boundaries of a notable laboratory abnormality should be evaluated by the investigator and additional evaluations should be performed if judged appropriate by the investigator. If the laboratory abnormality is the primary reason for an unforeseen hospitalization or otherwise fulfills the criteria for a Serious Adverse Event, then the procedure for notification of serious adverse events must be followed. Likewise, if the laboratory abnormality leads to discontinuation from the study or from treatment, then the patient must be followed until the abnormality resolves or until it is judged to be permanent.

1. Hematology

Hemoglobin, white blood cell count, lymphocyte and platelet count will be measured at every visits at 3-monthly intervals until the end of the stud.

2. Blood chemistry

Blood chemistry: serum creatinine, urea nitrogen, uric acid, total carbon dioxide, potassium, sodium and chlorine, alanine aminotransferase, aspartate aminotransferase, albumin, total bilirubin, triglyceride, cholesterol, very low density lipoprotein, high density lipoprotein, low density lipoprotein, calcium, phosphorus, glucose. It is necessary to remind the patient at the first visit that blood should be taken on a fasting status at the next visit.

3. Urinary analysis

A qualitative microscopic determination - white blood cells per high power field (WBCs/HPF) and red blood cells per high power field (RBCs/HPF) will be performed at each visit.

4. 24-hour urine protein excretion rate

24-hour urine collection for protein excretion will be performed at every visit.

5. Scoring of histological lesions

The renal biopsy will receive at least immunomicroscopy (Immunohistochemistry or Immunofluorescence) and lightmicroscopy. The renal biopsy material or electronic images with PAS (periodic acid Schiff) stain will be collected. The histological lesions will be reviewed at Visit 5 and graded according to the Oxford Classification by two independent renal pathologists.

7.11 Data management

A trained study staff will be selected and assigned for EDC system development. The CRF will be part of the EDC system which allows documentation of all variables and covariates in a standardized way. Detailed information on data management, including procedures for data collection, retrieval and preparation will be given in the Data Management Plan (DMP). DMP and information on the EDC system will be kept as stand-alone documents.

1. Prior and concomitant medication

Any diagnoses/diseases/event terms documented in the following forms will be coded using the latest Medical Dictionary for Regulatory Activities (MedDRA) version:

2. Co-morbidities (medical history, concomitant diseases)
3. Adverse events

7.12 Data analysis

Statistical considerations

All statistical details including calculated variables and proposed format and content of tables will be detailed in the Statistical Analysis Plan (SAP). The SAP will be finalized before study database lock. The SAP will be kept as a stand-alone document.

Analysis sets

1. ITTs (Intention-to-test set): All enrolled subjects who sign the informed consent and undergo randomization. ITTs will be used for all efficacy analyses
2. SS (Safety set): All enrolled patients who have used the study drug. The SS set will serve as the primary analysis population for all safety outcomes.

Subgroup analysis

The followings are possible subgroup factors currently considered, including, but not limited to:

1. Different age groups
2. Gender subgroup
3. Hypertensive vs. non-hypertensive patients
4. 24-hour urinary protein excretion (<1.0g/day, ≥1.0g/day)
5. Renal function (eGFR<50 or ≥50ml/min per $1.73m^2$)
6. Histological lesion scoring (E1, E0; C0, C1, C2; T0, T1, T2)

7.13 Quality control

Data quality

Before study start at the sites, all investigators and other involved site personnel will be sufficiently trained on the background and objectives of the study and ethical as well as regulatory obligations. Investigators and site personnel will have the chance to discuss and develop a common understanding of the protocol and the CRF. They will be trained on the importance to ensure that all relevant study data entered in the EDC should be retrievable from the patient's medical records.

A group of trained staff will be selected and assigned for EDC system development, quality control, verification of the data collection, data analysis.

All observations will be recorded in a standardized CRF. After data entry, missing or implausible data will be queried, and the data will be validated. A check for multiple documented patients will be done.

Detailed information on checks for completeness, accuracy, plausibility and validity are given in the Data Management Plan (DMP).

Medical Review of the data will be performed according to the Medical Review Plan (MRP). The purpose of the Medical Review is to verify the data from a medical perspective for plausibility, consistency, and completeness and to identify potential issues that could affect the robustness of the collected study data or the progress of the study. Detailed information on the Medical review will be described in the MRP.

DMP, MRP and validation documentation are kept as stand-alone documents.

Quality review

Quality review will follow a sequential, risk-based approach: in a first step the site's training status will be assessed via standardized (telephone) interviews.

In a second step, source data verification (SDV) will be conducted. The purpose of the SDV is to review the documented data for completeness and plausibility, adherence to the OS protocol and verification with source documents. To accomplish this, reviewers will access patient records onsite for data verification.

Detailed measures for quality review activities are described in the Quality Review Plan (QRP). The QRP is kept as stand-alone document.

Storage of records and archiving

All relevant documents of this study will be stored after the end or discontinuation of the study for at least 25 years. Any data as well as programs from statistical programming performed to generate results will be stored within the programming system for at least 25 years.

Participating study sites are required to archive and retain study documents for the period stipulated by local regulations, considering possible audits and inspections from local authorities.

8 Protection of human subjects

Ethical conduct of the study

Before the start of the study, the protocol, informed consent document, any proposed advertising material and any other appropriate documents will be submitted to the appropriate Human Research Ethics Committee (HREC) for written approval. The investigator will submit and, where necessary, obtain approval from the above parties for all subsequent and substantial amendments to the original approved documents. Study medication can only be supplied to the investigator after documentation on all ethical and regulatory requirements for starting the study has been received by the Trial Coordinating Centre. Safety reports, annual progress reports and a final report at conclusion of the trial will be submitted to the Regulatory Authorities, research ethics committees and if applicable, to the study treatment manufacturer within the timelines defined in the Regulations.

Independent ethics committee (IEC) or institutional review board (IRB)

In all countries where reference to an IEC/IRB is required, documented approval from appropriate IECs/IRBs will be obtained for all participating centers prior to study start. When necessary, an

extension, amendment or renewal of the IEC/IRB approval must be obtained and also forwarded to the study initiator and funder. The IEC/IRB must supply to the study initiator and funder, upon request, a list of the IEC/IRB members involved in the vote and a statement to confirm that the IEC/IRB is organized and operates according to applicable laws and regulations.

Patient information and consent

Before documentation of any data, informed consent is obtained from the patient by authorized site personnel. In countries where required by law or regulation, the investigator must have the IECs/IRB written approval/favorable opinion of the informed consent form and any other information to be provided to patients prior to the beginning of the observation.

Confidentiality

All investigators ensure adherence to applicable data privacy protection regulation. Data are transferred in encoded form only. The entire documentation made available does not contain any data which, on its own account or in conjunction with other freely available data, can be used to re-identify natural persons. The investigators are obligated to ensure that no documents contain such data.

All records identifying the patient will be kept confidential and will not be made publicly available. Patient names will not be supplied. If the patient name appears on any document, it must be obliterated before a copy of the document is supplied. Study findings stored on a computer will be stored in accordance with local data protection laws.

The investigator will maintain a list to enable patients' records to be identified in case of queries. In case of a report of a serious adverse event (SAE), the responsible pharmacovigilance person may ask for additional clarification. In that case, other people is not allowed to directly contact the patient. All additional information will be provided by the investigator.

9 Management and reporting of adverse events/adverse reactions

Definitions

Adverse event (AE)

Any untoward medical occurrence in a patient with the use of a medical product and which does not necessarily have to have a causal relationship (association) with this product. An AE can therefore be any unfavorable and unintended sign (including an abnormal laboratory finding), symptom, or disease temporally associated with the use of the product, whether or not related to the product.

Adverse Reaction (AR)

Adverse reaction is defined as a response to a medical product which is noxious and unintended. An AR is any AE judged as having a reasonable suspected causal relationship to a medicinal product.

Causal relationship

The assessment of the causal relationship between an AE and the use of the study product is a clinical decision by the investigator based on all available information at the time of the completion of the CRF. The assessment is based on the question whether there was a "reasonable causal relationship" to the study product in question. Possible answers are "yes" or "no".

An assessment of "no" would include:

1. The existence of a clear alternative explanation
2. Non-plausibility

   An assessment of "yes" indicates that there is a reasonable suspicion that the AE is associated with the use of the study product. Factors to be considered in assessing the relationship of the AE to study product include:

3. The temporal sequence from the use of product: The event should occur after the product is given. The length of time from product exposure to event should be evaluated in the clinical context of the event.
4. Recovery on product discontinuation (de-challenge), recurrence on product re-introduction (re-challenge): Subject's response after de-challenge or subject’s response after re-challenge should be considered in the view of the usual clinical course of the event in question.
5. Underlying, concomitant, intercurrent diseases: Each event should be evaluated in the context of the natural history and course of the disease being treated and any other disease the subject may have.
6. Concomitant medication or treatment: The other products the subject is taking or the treatment the subject receives should be examined to determine whether any of them may be suspected to cause the event in question.
7. Spontaneous case reports from consumers or health care professionals are generally considered to be adverse reactions, since the act of reporting a case is assumed to imply a judgment of possible causality on the part of the reporter, unless there is a clear statement from the reporter that a causal relationship can be excluded.

Serious adverse event/serious adverse reaction

An adverse event (AE) or adverse reaction (AR) is serious if it:

2. results in death,
3. is life-threatening
4. requires inpatient hospitalization or prolongation of existing hospitalization.

   A hospitalization or prolongation of hospitalization will not be regarded as a serious adverse event (SAE) if at least one of the following exceptions is met: (1) the admission results in a hospital stay of less than 12 hours; (2) the admission is pre-planned (i.e. elective or scheduled surgery arranged prior to the start of the study); (3) the admission is not associated with an AE (e.g. social hospitalization for purpose of respite car.

   However, it should be noted that invasive treatment during any hospitalization may fulfill the criterion of ‘medically important’ and as such may be reportable as an SAE dependent on the clinical judgment. In addition, where local regulatory authorities specifically require a more stringent definition, the local regulation takes precedence;

5. results in persistent or significant disability or incapacity
6. is a congenital anomaly, birth defect or fetal mental impairment
7. is medically significant:

o Medical and scientific judgment should be exercised in deciding whether an AE/AR may be considered serious (due to an important medical event) because it jeopardizes the health of the

patient or may require intervention to prevent another serious condition (death, a life-threatening condition, hospitalization or persistent or significant disability).

- Examples of such events are:
  - invasive treatment during any hospitalization, in an emergency room or at home for allergic bronchospasm
  - blood dyscrasia or convulsions that do not require hospitalization
  - development of any drug dependency or abuse
  - lack of drug effect if the product was used for the treatment of acute life-threatening diseases (apply medical judgment) or reports of any transmission of an infectious agent via a product

Investigational medical product

1. Medical product being assessed for safety or performance in a clinical investigation
2. NOTE: This includes medical products already on the market that are being evaluated for new intended uses, new populations, new materials or design changes.

Collection

After enrollment into the study, all non-serious adverse events (AE) must be documented on the AE Report Form or in the CRF/EDC system and forwarded to the sponsor within 7 calendar days of awareness. All serious AEs (SAE) must be documented and forwarded immediately (within one business day of awareness). For each AE, the investigator or a delegate must assess and document the seriousness, duration, relationship to product, action taken and outcome of the event.

If a pregnancy occurs during the study, although it is not a serious adverse event itself, it should be documented and forwarded to the sponsor within the same time limits as a serious adverse event. The result of a pregnancy will be followed-up according to applicable SOPs. Any data on abnormal findings concerning either the mother or the baby will be collected as adverse events.

All drug related events must be documented on the product related CRF form or in the CRF / EDC system and also on the PTC form(s). Incidents must be forwarded to the study initiator and funder within 24 hours (immediately) and other events must be forwarded within 7 calendar days of awareness. Products suspected of defects should be retained for recycling and returned to the sponsor for further inspection and verification

The documentation of any AE/SAE/drug related events ends with the completion of the observation period of the patient. However, any AE/SAE - regardless of the relationship and the seriousness - occurring within 30 $\pm$ 7 days after the last use of MMF within the study period has to be documented and forwarded to the sponsor within the given timelines, even if this period goes beyond the end of observation.

As long as the patient has not used any MMF within the frame of the study AEs /SAEs do not need to be documented as such in this observational study. However, they are part of the patient's medical history.

For any serious product-related AE occurring after study end, the standard procedures that are in place for spontaneous reporting has to be followed.

Management and reporting

Non-serious AEs

The outcome of all reported AEs will be followed up and documented. Where required, investigators might be contacted directly by the responsible study staff to provide further information.

Non-serious ARs

For non-serious ARs occurring under non-MMF the investigator has to account for and comply with the reporting system of the product's Marketing Authorization Holder within the frame of local laws and regulations as well as other locally applicable laws and regulations.

Serious AEs

Any SAE or pregnancy entered into the CRF/EDC system will be forwarded immediately by the EDC system (or alternatively the study staff manually within one business day of awareness) to the pharmacovigilance country head being responsible for SAE processing. The outcome of all reported SAEs (resolution, death etc.) will be followed up and documented. Where required, investigators might be contacted directly by the pharmacovigilance country head in charge to provide further information.

For SAEs that occurred while administering non-MMF the investigator has to account for and comply with the reporting system of the product's Marketing Authorization Holder within the frame of local laws and regulations as well as other locally applicable laws and regulations.

Incidents

All drug-related events will be forwarded to the pharmacovigilance staff being responsible for product event processing. Product technical complaints not related to an AE/SAE will be forwarded to the relevant quality department. The outcome of all reported incidents (resolution, death etc.) will be followed up and documented. Where required, investigators might be contacted directly by the pharmacovigilance staff in charge to provide further information.

For any MMF related events occurring after study end, the standard procedures that are in place for spontaneous reporting has to be followed.

According to relevant local regulations, AE/SAE/medical product events related to the use of medical product and product defects that may cause serious adverse events should be submitted by the medical product prescription-provide institution to the relevant regulatory authority within the prescribed time limit.

Evaluation

Whenever new important safety information is received, e.g. case reports from an investigator, the reports are processed and entered into the pharmacovigilance safety database. These reports will be reviewed on a regular basis (for information on collection, management and reporting of case reports, refer to 0 and 0). If a potential safety signal is suspected, an investigation of the suspected potential signal will be performed according to internal standard operating procedures, for further evaluation within the context of benefit risk.

10 References

1. Pozzi C, Bolasco PG, Fogazzi GB, Andrulli S, Altieri P, Ponticelli C, Locatelli F. Corticosteroids in IgA nephropathy: a randomised controlled trial. *Lancet.* 1999; 353: 883-887.

2. Moldoveanu Z, Wyatt RJ, Lee JY, Tomana M, Julian BA, Mestecky J, et al. Patients with IgA nephropathy have increased serum galactose-deficient IgA1 levels. *Kidney Int.* 2007;71:1148-1154.

3. Suzuki H, Fan R, Zhang Z, Brown R, Hall S, Julian BA, et al. Aberrantly glycosylated IgA1 in IgA nephropathy patients is recognized by IgG antibodies with restricted heterogeneity. *J Clin Invest*. 2009;119:1668-1677.

4. Floege J, Eitner F. Current therapy for IgA nephropathy. *J Am Soc Nephrol*. 2011; 22: 1785-1794.

5. Kidney Disease: Improving Global Outcomes (KDIGO) Glomerulonephritis Work Group. KDIGO Clinical Practice Guideline for Glomerulonephritis. *Kidney Inter Suppl*. 2012; 2:139-274.

6. Allison AC, Eugui EM. Purine metabolism and immunosuppressive effects of mycophenolate mofetil (MMF). *Clin Transplant.* 1996;10:77-84.

7. Tang S, Leung JCK, Chan LYY, Lui YH, Tang CSO, Kan CHIH, et al. Mycophenolate mofetil alleviates persistent proteinuria in IgA nephropathy. *Kidney Int*. 2005; 68: 802-812.

8. Tang SC, Tang AW, Wong SS, Leung JC, Ho YW, Lai KN. Long-term study of mycophenolate mofetil treatment in IgA nephropathy. *Kidney Int*. 2010; 77: 543-549.

9. Hou FF, Xie D, Zhang X, Chen PY, Zhang WR, Liang M, Guo ZJ, Jiang JP. Renoprotection of Optimal Antiproteinuric Doses (ROAD) Study: a randomized controlled study of benazepril and losartan in chronic renal insufficiency. *J Am Soc Nephrol*. 2007; 18: 1889-1898.

Statistical Analysis Plan

**MAIN** / The Effect of **M**ycophenolate Mofetil on Renal Outcomes in **A**dvanced **I**mmunoglobulin A **N**ephropathy			
**Study Drug**	Mycophenolate Mofetil		
**Study Purpose:**	To access the effect of mycophenolate mofetil plus losartan compared with lorsatan alone on clinically important renal outcomes		
**Clinicaltrials.gov study number:**	NCT01854814	**Protocol Version/Date:**	V2.0/15 Nov 2017
**SAP Version, Date:**	V1.0, 15 Mar 2022	**SAP includes TLF specification:**	yes<br>no ☑
**Author:**	Xin Xu, Di Xie, Fan Fan Hou		

Table of Contents

1. Introduction 4
1.1. Background 4
1.2. Protocol Version and Amendments 4
2. The Study Objectives 4
3. Study Design 4
3.1. Study Population 5
3.2. Study Drug 6
3.3. Treatment Groups 6
3.4. Primary Outcomes 6
3.6. Secondary Outcomes 6
3.7. Safety Endpoints 6
3.7.1. Adverse Events 6
3.7.2. Laboratory Examination 7
3.8. Visits 7
4. General Statistical Considerations 9
4.1. General Principles 9
4.2. Handling of Lost to Follow Up and Premature Discontinuation 9
4.3. Handling of Missing Data 9
4.4. Multiplicity Adjustment 9
5. Analysis Sets 10
5.1. Intention-to-test Set (ITTs) 10
5.2. Safety Set (SS) 10
6. Statistical Analyses 10
6.1. Population Characteristics at Randomization 10
6.2. Distribution of the specified clinical variables at each visit 11
6.3. Efficacy Analyses 11
6.3.1. Main Analyses of the Primary Outcomes 11
6.3.2. Subgroup Analyses of the Primary Outcomes 11
6.3.3. Analysis of the Secondary Outcomes 11
6.3.3.1. The Time to 30% Reduction in eGFR 11
6.3.3.2. Annual Absolute Change in eGFR (eGFR Slope) 12
6.3.3.3. Rapid Renal Function Decline (eGFR Slope < -5 ml/min/1.73m^2/year) 12
6.3.3.4. Percentage Changes in the Rate of Urinary Protein Excretion Rate 12
6.4. Safety Analysis 12
6.4.1. Adverse events 12
6.4.2. Laboratory Examination 13
7. Document History and Changes in the Planned Statistical Analysis 13
8. References 13

Abbreviations

AE	Adverse Event
ANOVA	Analysis of Variance
AR	Adverse Reaction
BMI	Body Mass Index
DBP	Diastolic Blood Pressure
eGFR	Estimated Glomerular Filtration Rate
ESRD	End Stage Renal Disease
HPF	High Power Field
HR	Hazard Ratio
ID	Identification
IgA	Immunoglobulin A
ITTs	Intention-to-Test Set
KDIGO	Kidney Disease Improving Global Outcomes
LOCF	Last Observation Carry Forward
MAP	Mean Arterial Pressure
MMF	Mycophenolate Mofetil
PI	Principal Investigator
RAS	Renin-Angiotensin System
RBCs	Red Blood Cells
SAE	Serious Adverse Event
SAP	Statistical Analysis Plan
SBP	Systolic Blood Pressure
SD	Standard Deviation
SOP	Standard Operating Procedure
SS	Safety Set
TLF	Tables, Listings and Figures
UPE	Urine Protein Excretion
WBCs	White Blood Cells

1. Introduction

1.1. Background

Immunoglobulin A (IgA) nephropathy is the most common type of glomerulonephritis. Up to 40% of the patients with IgA nephropathy progress to end stage renal disease (ESRD) within 20 years, making it the leading cause of kidney failure in many parts of the world.[1]

The effective treatment for IgA nephropathy remains uncertain. The central role of the immune and autoimmune activation in the pathogenesis of IgA nephropathy[2,3] indicates a potential benefit of immunosuppression for treating the disease. However, the efficacy of immunosuppression in IgA nephropathy has been controversal.[1,4] Current management of IgA nephropathy remains focused on non-immunosuppression-based strategies, i.e. supportive care, to reduce proteinuria and slow the disease progression. This encompasses optimal inhibition of renin-angiotensin system (RAS), rigorous blood pressure control, and lifestyle modification.[4] However, despite intensive supportive care, considerable number of patients still have massive proteinuria and remain at high risk of disease progression. The Kidney Disease Improving Global Outcomes (KDIGO) guidelines suggest the use of systemic glucocorticoids in patients with a proteinuria level of ≥1g/day and an estimated glomerular filtration rate (eGFR) of ≥50ml/min/1.73m^2, despite of 3 to 6 months of optimized supportive care.[5] The benefits of immunosuppressive therapy, particularly when added to supportive care, remain largely unclear.

Mycophenolate mofetil (MMF) is a potent immunosuppressive agent selective for lymphocytes and inhibits antibody production by B cells more than any other immunosuppressants.[6] However, previous reports on the efficacy of MMF treatment in patients with IgA nephropathy are controversial, probably due to the small sample size in most published studies, and differences in patients characteristics, e.g. low or high risk of disease progression, and with or without consistent blockade of RAS. [5, 7, 8]

The present study is designed to test the hypothesis that addition of MMF to the comprehensive supportive care would be superior to the supportive care alone in reducing the risk of clinically important kidney outcomes in patients with IgA nephropathy who are at a high risk of disease progression.

1.2. Protocol Version and Amendments

This statistical analysis plan (SAP) is based on the protocol "The Effect of **M**ycophenolate Mofetil on Renal Outcomes in **A**dvanced **I**mmunoglobulin A **N**ephropathy" (**Clinicaltrials.gov study number:** NCT01854814, Protocol version: V2.0, Version date: 15 Nov 2017).

2. The Study Objectives

The primary objective of the study is to test if combination of MMF and losartan is more effective than losartan alone in reducing the risk of disease progression in patients with IgA nephropathy.

3. Study Design

This study is a prospective, open-label, randomized controlled, blinded endpoint (PROBE) trial. This trial will include participants with IgA nephropathy who are at a high risk of disease progression.

Patients with IgA nephropathy were recruited at the renal division of Nanfang Hospital, Southern Medical University. After recruitment, the patients first entered into a 12-week run-in period, during which the patients were treated with comprehensive supportive care, including blockade of RAS using maximum tolerable daily dose of losartan to reduce blood pressure to a target below 130/80 mmHg and a urine protein excretion (UPE) below 0.75g per day[9]. The participants were also advised for lifestyle modification, including quitting smoking, restricting high-salt intake, and avoiding nephrotoxic drugs. Anemic patients with a hemoglobin level lower than 110g/L were treated with erythropoietin. Statin was used when necessary. Dietary sodium intake were monitored by urinary sodium excretion rate (or chloride excretion rate in patients treated with sodium bicarbonate) measured by 24-hour urine sample.

The patients who were tolerable to losartan (<30% eGFR decline and without hyperkalemia) and proteinuria (0.75-3.5 g/day) at the end of run-in period were randomized to receive MMF plus supportive care or supportive care alone. The MMF plus supportive care group received a daily oral dose of 1.5g MMF for the first 12 months, followed by a maintenance daily dose of 0.75g to 1.0g for at least 6 months. Both MMF plus supportive care and supportive care alone group stayed on supportive care as in the run-in period. All participants were followed up at regular intervals for 3 years.

3.1. Study Population

The target population is the patients with IgA nephropathy who are at a high risk of disease progression.

Inclusion criteria for entering run-in period:

- Adult female or male (18 years of age or older);
- Biopsy-proven primary IgA nephropathy;
- 24 hour urinary proteinuria excretion ≥1g; and eGFR <60 ml/min/1.73m^2, or persistent hypertension, defined as blood pressure ≥140/90 mmHg in two visits at least one day apart or need of antihypertensive drug;
- Signed informed consent

Exclusion criteria for entering run-in period:

- Familial IgA nephropathy
- Concomitant disease: cancer, infection, diabetes mellitus, connective tissue disease, abnormal liver function, or liver disease;
- Pregnant women;
- eGFR<30 ml/min/1.73m^2;
- Prior immunosuppressive therapy;
- Severe hypertension (systolic blood pressure over 180 mmHg and/or diastolic blood pressure over 110 mmHg);
- Serum albumin level < 30g/L;
- Hyperkalemia (blood potassium ≥ 5.5 mmol/l);
- Inability to comply with study and follow-up procedures.

Inclusion criteria for randomization:

UPE remained at 0.75-3.5 g per day at the end of run-in period

Exclusion criteria for randomization for randomization:

Not tolerable to RAS blockade (eGFR decline over 30% of baseline or developing hyperkalemia) during run-in phase

3.2. Study Drug

Drug information

- Drug: Mycophenolate mofetil
- Formulation: Mycophenolate mofetil tablets 0.25g/tablet
- Manufacturer: Hangzhou ZhongmeiHuadong Pharmaceutical Co., Ltd

3.3. Treatment Groups

All participants meeting the inclusion and the exclusion criteria were randomized to either MMF plus the supportive care group or the supportive care alone in a 1:1 ratio. Both the participants and the care personnels were aware of treatment assignment. However, the treatment assignments were not disclosed to the members of the endpoint adjudicating committee and the statistical analysis group.

3.4. Primary Outcomes

There are two primary outcomes.

1. The time to the first event of the composite outcomes, including:
   a doubling of serum creatinine, defined as serum creatinine doubled over the baseline and confirmed by another measurement at least four weeks apart; or
   onset of end stage kidney disease, defined by the need for maintenance dialysis or renal transplantation or kidney failure (defined as eGFR <15 ml/min/1.73m^2 and need for dialysis or renal transplant, but renal replacement therapy is not available or patients refuse to receive renal replacement therapy); or death due to kidney or cardiovascular cause.

2. The time to progression of CKD, defined by a sustained ≥30% decrease in eGFR and to a level of <60 ml/min/1.73m^2 if the baseline eGFR was ≥60 ml/min/1.73m^2, or ≥50% decrease in eGFR if the baseline eGFR was <60 ml/min/1.73m^2. The CKD progression endpoints were also verified by another measurement at least four weeks apart.

3.6. Secondary Outcomes

There are four secondary outcomes.

1) The time to 30% reduction in eGFR from the baseline.
2) Annual absolute changes in the eGFR (eGFR slope) over the trial period.
3) Rapid eGFR decline (<-5 ml/min/1.73m^2/year).
4) Percentage change in the rate of urinary protein excretion at the end of trial over the baseline.

3.7. Safety Endpoints

3.7.1. Adverse Events

(1) AEs/SAEs

AEs/SAEs occurred during the study period from the first application of MMF to 30±7 days after the last use of MMF will be analyzed and summarized.

Adverse event (AE): Any untoward medical occurrence in a patient with the use of a medical product and which does not necessarily have to have a causal relationship (association) with this product. An AE can therefore be any unfavorable and unintended sign (including an abnormal laboratory finding), symptom, or disease temporally associated with the use of the study device, whether or not related to the product.

Adverse Reaction (AR): is defined as a response to the study device which is noxious and unintended. An AR is any AE judged as having a reasonable suspected causal relationship to the study device. The assessment of the causal relationship between an AE and the use of the study device is a clinical decision by the investigator based on all available information at the time of the completion of the CRF. The assessment is based on the question whether there was a "reasonable causal relationship" to the study device in question. Possible answers are "yes" or "no".

Serious adverse event/serious adverse reaction: an adverse event (AE) or adverse reaction (AR) is serious if it:

- results in death,
- is life-threatening
- requires inpatient hospitalization or prolongation of existing hospitalization.
- results in persistent or significant disability or incapacity
- is a congenital anomaly, birth defect or fetal mental impairment
- is medically significant: Medical and scientific judgment should be exercised in deciding whether an AE/AR may be considered serious (due to an important medical event) because it jeopardizes the health of the patient or may require intervention to prevent another serious condition (death, a life-threatening condition, hospitalization or persistent or significant disability).

3.7.2. Laboratory Examination

Lab Examination during the screening visit are as following.

- Blood hematology: counts of red blood cells, white blood cells, neutrophil count, lymphocyte count, platelets, hemoglobin
- Blood biochemistry: serum creatinine, urea nitrogen, uric acid, total carbon dioxide, potassium, sodium and chlorine, alanine aminotransferase, aspartate aminotransferase, albumin, total bilirubin, triglyceride, cholesterol, very low density lipoprotein, high density lipoprotein, low density lipoprotein, calcium, phosphorus, fasting glucose
- Routine urinal assays: urine protein, white blood cells per high power field (WBCs/HPF) and red blood cells per high power field (RBCs/HPF)
- 24-hour UPE rate
- Scoring of histological lesions

3.8. Visits

Information to be collected at the visits are summarized in Table 1.

Table 1: Tabulated overview on data collected during the study

Phase	Screening	Run-in				Treatment period																						
**Visits**	**1**	**2**	**3**	**4**	**5**	**6**	**7**	**8**	**9**	**10**	**11**	**12**	**13**	**14**	**15**	**16**	**17**	**18**	**19**	**20**	**21**	**22**	**23**	**24**	**25**	**26**	**27**	**28**
**Time (week)**	**-14**	**-12**	**-8**	**-4**	**0**	**4**	**8**	**12**	**16**	**20**	**28**	**36**	**44**	**52**	**60**	**68**	**76**	**78**	**86**	**94**	**102**	**110**	**118**	**126**	**134**	**142**	**150**	**156**
Informed consent form		×																										
In/exclusion criteria		×																										
Medical history/ demography	×																											
Height, weight	×	×	×	×	×	×	×	×	×	×	×	×	×	×	×	×	×	×	×	×	×	×	×	×	×	×	×	×
Physical examination	×	×			×																							×
Vital signs	×	×	×	×	×	×	×	×	×	×	×	×	×	×	×	×	×	×	×	×	×	×	×	×	×	×	×	×
Screening log	×																											
Randomization					×																							
Blood chemistry [1]	×		×		×	×	×	×	×	×	×	×	×	×	×	×	×	×	×	×	×	×	×	×	×	×	×	×
Hematology	×		×		×	×	×	×	×	×	×	×	×	×	×	×	×	×	×	×	×	×	×	×	×	×	×	×
Urinary analysis	×		×		×	×	×	×	×	×	×	×	×	×	×	×	×	×	×	×	×	×	×	×	×	×	×	×
24-hour urine protein [2]	×		×		×	×	×	×	×	×	×	×	×	×	×	×	×	×	×	×	×	×	×	×	×	×	×	×
Diet education	×	×	×	×	×	×	×	×	×	×	×	×	×	×	×	×	×	×	×	×	×	×	×	×	×	×	×	×
Study drug dispensation						×	×	×	×	×	×	×	×	×	×	×	×	×										
Concomitant medications	×	×	×	×	×	×	×	×	×	×	×	×	×	×	×	×	×	×	×	×	×	×	×	×	×	×	×	×
AEs/SAEs			×	×	×	×	×	×	×	×	×	×	×	×	×	×	×	×	×	×	×	×	×	×	×	×	×	×
Endpoints						×	×	×	×	×	×	×	×	×	×	×	×	×	×	×	×	×	×	×	×	×	×	×

1. Including: serum creatinine (CR), urea nitrogen (BUN), uric acid (UA), total carbon dioxide (tCO2), potassium (k), sodium (NA) and chlorine (CL), alanine aminotransferase (ALT), aspartate aminotransferase (AST), albumin (ALB), total bilirubin (T-Bil), triglyceride (TG), cholesterol (Chol), very low density lipoprotein (VLDL), high density lipoprotein (HDL), low density lipoprotein (LDL), calcium (CA), phosphorus (P), glucose (Glu). It is necessary to remind the patient at the first visit that blood should be taken on a fasting status at the next visit.

2. It is necessary to remind the patient to take a 24-hour urine sample at the next visit at the previous visit

4. General Statistical Considerations

4.1. General Principles

The analysis conducted will follow the relevant standard operating procedures (SOP). Programs, logs, and output will be reviewed for accuracy according to the SOPs for programming and quality control. Descriptive analysis of the data will be performed using summary statistics for categorical and quantitative (continuous) data. Continuous data will be described by the number of non-missing values, median, mean, standard deviation, minimum, and maximum as well as lower and upper quartiles. If not specified, the mean and median are retained 1 decimal place more than the original data, and the standard deviation is retained 1 decimal place more than the mean, but all statistics are rounded to no more than 3 decimal places. Frequency tables (the number of cases, percentages rounded to retain 1 decimal place) will be generated for categorical data. Selected continuous variables will be categorized in a clinically meaningful way or by quartiles of the distribution. In addition, if sparse cells (i.e. count<10 for variables) are identified during the analysis, the sparse cells may be combined into other meaningful categories, excluded from analysis, or left as they are. Decisions will be made by the study team at the time sparse cells are identified.

4.2. Handling of Lost to Follow Up and Premature Discontinuation

The distribution of subjects will be presented including the number and percentage of subjects enrolled, screening failure, lost to follow up, discontinuation, and completing the study. For the subjects who failed screening, the number and percentage of subjects for each reason will be clarified and summarized, such as inclusion criteria not met / exclusion criteria met, patient decision, physician decision (medical reason, unable to participate / internal hospital guidelines) and others. The detailed reasons for screening failure of each subject will be presented in the lists.

For the subjects who are lost to follow up or prematurely discontinue the trial, their main reasons will be clarified and summarized, such as patient decision, physician decision, patient voluntary withdrawal, AE, other reasons. The detailed reasons for loss to follow up or premature discontinuation of each subject will be presented in the lists.

4.3. Handling of Missing Data

For primary and secondary endpoints, missing data will be treated as missing, and no imputations will be performed.

Counts of missingness will be reported in descriptive analysis of categorical variables, and percentages of the non-missing categories will be based on all values. For continuous variables, the number of non-missing values as well as missing values will be reported, but descriptive summaries will be based on the number of non-missing values.

4.4. Multiplicity Adjustment

For the primary analysis, a two-sided p value of <0.025 was regarded as statistically significant. For the secondary and safety analyses, no multi-test adjustment was employed, and a two-sided p value of <0.05 was regarded as statistically significant. The outcomes are clearly categorized by degree of importance (primary, secondary) and a limited number of subgroup analyses are pre-specified.

5. Analysis Sets

5.1. Intention-to-test Set (ITTs)

ITT includes all subjects randomized to treatment. The ITT set will be the set for all efficacy analyses.

5.2. Safety Set (SS)

SS is a subset of ITT. It consists of ITT participants excluding the ones without taking any study medication or having no record of follow up after randomization. SS will be used for the safety analysis.

6. Statistical Analyses

6.1. Population Characteristics at Randomization

The demographic and clinical characteristics of the ITT set at randomization stratified by the treatment group will be summarized. Summary statistics for quantitative variables will include N, mean, SD, or median, Q1, Q3. Summary statistics for qualitative variables will be N (%) for each level. Unless otherwise specified, the calculation of proportions will be based on the non-missing data. Difference in the variable distribution between the two treatment groups will be tested using the unpaired two-sample t-test (quantitative variables) and the chi-square test (qualitative variables). The following variables will be analyzed:

- Demography: age, sex, BMI.
- Comorbidity: smoking status, macrohematuria, history of hypertension, other reported medical history and co-morbidities.
- Vital Signs: body temperature (°C), pulse (beats/min), blood pressure (mmHg), respiratory rate.
- Use of Medications: anti-hypertensive drugs and types; statin.
- Baseline serum creatinine and eGFR. The baseline creatinine is calculated as the mean of the two creatinine values from Visit 4 (pre-randomization visit) and Visit 5 (randomization visit). Baseline eGFR is calculated from baseline serum creatinine using the CKD-EPI equation
- Other laboratory results:

  -- Hematology: hemoglobin, white blood cell count, lymphocyte and platelet count

  -- Blood chemistry: urea nitrogen, uric acid, total carbon dioxide, potassium, sodium and chlorine, alanine aminotransferase, aspartate aminotransferase, albumin, total bilirubin, triglyceride, cholesterol, very low density lipoprotein, high density lipoprotein, low density lipoprotein, calcium, phosphorus, glucose

  -- Urinary analysis: white blood cells per high power field (WBCs/HPF) and red blood cells per high power field (RBCs/HPF)

  -- 24-hour UPE

- Pathological features: percentage of glomeruli with global glomerulosclerosis, percentage of glomeruli with segmental glomerulosclerosis, mesangial hypercellularity, endocapillary hypercellularity, tubular atrophy/interstitial fibrosis, percentage of glomeruli with crescents; MEST-C Oxford Score.

6.2. Distribution of the specified clinical variables at each visit

To calculate and plot the group means and SDs of blood pressure (SBP, DBP, MAP), 24h urine protein excretion, and eGFR of the two treatment groups at each visit. LOCF will be used to fill the missing values.

6.3. Efficacy Analyses

6.3.1. Main Analyses of the Primary Outcomes

- The number of the composite outcome (primary outcome 1) and its components by each treatment group in ITT.
- The number of disease progression (primary outcome 2) by each treatment group in ITT.
- Kaplan-Meier curves of cumulative survival rates of the two primary outcomes by two treatment groups in ITT.
- To perform log-rank tests on the two primary outcomes between the two treatment groups in ITT. A p-value of 0.025 will be regarded as statistically significant.
- Estimate the hazard ratios (and 95% confidence intervals) of the treatment for the two primary outcomes, respectively, using the Cox proportional hazard models, with and without covariate adjustment. The adjusting covariates include age, sex, BMI, SBP, eGFR, logarithm of UPE rate and individual Oxford MEST-C scores at baseline.

6.3.2. Subgroup Analyses of the Primary Outcomes

For each of the two primary outcomes, Hazard ratios of the treatment in the following subgroups will be estimated using the Cox proportional hazard models, and differences in the HRs among the subgroups tested by the ANOVA test. The subgroups include:

(1) Age (<35 years, ≥35 years)
(2) Gender (male, female)
(3) Hypertensive (yes, no)
(4) baseline 24-hour UPE (<1.5 g/day, ≥1.5 g/day)
(5) baseline eGFR (<50 or ≥50ml/min /1.73m^2)
(6) Individual MESTC scores (M0, M1; E0, E1; S0, S1;C0, C1, C2; T0, T1, T2)

6.3.3. Analysis of the Secondary Outcomes

6.3.3.1. The Time to 30% Reduction in eGFR

- The number of patients reached 30% reduction in eGFR by each treatment group in ITT.
- Kaplan-Meier curves of cumulative survival rate of the outcome by two treatment groups in ITT.
- Perform log rank tests on the outcomes between the two treatment groups in ITT.

- Estimate the hazard ratios (and 95% confidence intervals) of the treatment using the Cox proportional hazard models, with and without covariate adjustment. The adjusting covariates include age, sex, BMI, SBP, eGFR, logarithm of UPE rate and individual Oxford MEST-C scores at baseline.

6.3.3.2. Annual Absolute Change in eGFR (eGFR Slope)

The eGFR slope of each participant in ITT during the trial period will be estimated using a linear mixed effect model with random intercept and random slope. The difference in the eGFR slope between the two treatment groups will be estimated and tested using a linear mixed effect model with an interaction term between treatment group and time.

6.3.3.3. Rapid Renal Function Decline (eGFR Slope < -5 ml/min/1.73m^2/year)

Tabulate the number of participants with an estimated eGFR slope < -5 ml/min/1.73m^2/year stratified by treatment group. Difference in the event rate between the treatment groups is tested using logistic regression analysis, with and without covariate adjustment. The adjusting covariates include age, sex, BMI, SBP, eGFR, logarithm of UPE rate and individual Oxford MEST-C scores at baseline. ORs and the confidence intervals will be reported.

6.3.3.4. Percentage Changes in the Rate of Urinary Protein Excretion Rate

The baseline 24-hour UPE is calculated as the mean of the two 24-hour UPE value from Visit 4 (pre-randomization visit) and Visit 5 (randomization visit). The 24-hour UPE at exit is calculated as the mean of from the last two visits before study exit. Percentage change is calculated as 100% x (exit value-baseline)/baseline. The difference in the percentage change between the two treatment groups will be tested using unpaired two-sample t-test.

6.4. Safety Analysis

The analysis of safety endpoints will be performed on the SS. For safety analysis, no formal hypothesis testing will be performed. Descriptive analysis will be performed to summarize safety events. Serious adverse events and adverse events will be summarized as the number of events and the number (%) of patients experiencing at least one event. Differences in the proportions of patients experiencing at least one event will be tested using Fisher’s exact test.

6.4.1. Adverse events

The number, frequency and incidence of the following AEs will be summarized,

1. AEs with special concern include drug-related AEs listed by the pharmaceutical company of the study drug, including leukopenia or leukocytosis, sepsis, gastrointestinal symptoms (diarrhea, stomachache, vomiting, anepithymia), certain types of repeated infections (pneumonia, influenza syndrome, urinary tract infection, herpes zoster, etc.), hepatic dysfunction or transaminase elevation, malignancy.
2. SAEs, study drug-related SAEs, and AEs leading to study withdrawal.

6.4.2. Laboratory Examination

Lab examination includes blood hematology, blood biochemistry, urine analysis during the screening visit. Descriptive analysis will be performed to summarize the results and clinical significance judgment of lab examination. For blood hematology, blood biochemistry, urine analysis, listings of patients with clinical significance results will be provided. The listings will be presented according to the subject ID, sex, age, date of test, examination index, examination result and/or clinical evaluation.

All measures will be summarized by use of standard measures of central tendency and dispersion using mean and standard deviation as well as quartile points at 0.25, 0.5 and 0.75 where appropriate stratified by measurement time points and by treatment group. Longitudinal mean plots will be used to display means and 95% confidence bands over time by randomized group. To assess the treatment effect on laboratory variables, a linear mixed effects model with a random intercept by subject and with treatment, time (categorical) and a treatment by time interaction as fixed effects will be used. The effect of the treatment will be assessed as the adjusted mean difference and its 95% confidence interval.

7. Document History and Changes in the Planned Statistical Analysis

None.

8. References

1. Pozzi C, Bolasco PG, Fogazzi GB, Andrulli S, Altieri P, Ponticelli C, Locatelli F. Corticosteroids in IgA nephropathy: a randomised controlled trial. Lancet. 1999; 353: 883-887.

2. Moldoveanu Z, Wyatt RJ, Lee JY, Tomana M, Julian BA, Mestecky J, et al. Patients with IgA nephropathy have increased serum galactose-deficient IgA1 levels. Kidney Int. 2007; 71: 1148-1154.

3. Suzuki H, Fan R, Zhang Z, Brown R, Hall S, Julian BA, et al. Aberrantly glycosylated IgA1 in IgA nephropathy patients is recognized by IgG antibodies with restricted heterogeneity. J Clin Invest. 2009; 119: 1668-1677.

4. Floege J, Eitner F. Current therapy for IgA nephropathy. J Am Soc Nephrol. 2011; 22: 1785-1794.

5. Kidney Disease: Improving Global Outcomes (KDIGO) Glomerulonephritis Work Group. KDIGO Clinical Practice Guideline for Glomerulonephritis. Kidney Inter Suppl. 2012; 2:139-274.

6. Allison AC, Eugui EM. Purine metabolism and immunosuppressive effects of mycophenolate mofetil (MMF). Clin Transplant. 1996; 10: 77-84.

7. Tang S, Leung JCK, Chan LYY, Lui YH, Tang CSO, Kan CHIH, et al. Mycophenolate mofetil alleviates persistent proteinuria in IgA nephropathy. Kidney Int. 2005; 68: 802-812.

8. Tang SC, Tang AW, Wong SS, Leung JC, Ho YW, Lai KN. Long-term study of mycophenolate mofetil treatment in IgA nephropathy. Kidney Int. 2010; 77: 543-549.

9. Cheung AK, Rahman M, Reboussin DM, Craven TE, Greene T, Kimmel PL, Cushman WC, Hawfield AT, Johnson KC, Lewis CE, Oparil S, Rocco MV, Sink KM, Whelton PK, Wright JT Jr, Basile J, Beddhu S, Bhatt U, Chang TI, Chertow GM, Chonchol M, Freedman BI, Haley W, Ix JH, Katz LA, Killeen AA, Papademetriou V, Ricardo AC, Servilla K, Wall B, Wolfgram D, Yee J; SPRINT Research Group. Effects of Intensive BP Control in CKD. *J Am Soc Nephrol.* 2017; 28: 2812-2823.
