# Supplementary material for: Effectiveness of Mycophenolate Mofetil Among Patients With Progressive IgA Nephropathy: A Randomized Clinical Trial
Source: JAMA Netw Open. 2023 Feb 6;6(2):e2254054. doi: 10.1001/jamanetworkopen.2022.54054 (PMC12578496; doi:10.1001/jamanetworkopen.2022.54054)
Supplement: Supplement 2. — eTable 1. Secondary Outcomes eTable 2. Summary of Major Adverse Events eFigure. Mean Estimated Glomerular Filtration Rate and Changes in Urinary Protein Excretion in Trial and Posttrial Phases [file jamanetwopen-e2254054-s002.pdf]

Supplemental Online Content

Hou FF, Xie D, Wang J, et al; MAIN Trial Investigators. Effectiveness of mycophenolate mofetil among patients with progressive IgA nephropathy: a randomized clinical trial. *JAMA Netw Open*. 2023;6(2):e2254054.
doi:10.1001/jamanetworkopen.2022.54054

eTable 1. Secondary Outcomes

eTable 2. Summary of Major Adverse Events

eFigure. Mean Estimated Glomerular Filtration Rate and Changes in Urinary Protein Excretion in Trial and Posttrial Phases

This supplemental material has been provided by the authors to give readers additional information about their work.

© 2023 Hou FF et al. *JAMA Network Open.*

eTable 1. Secondary Outcomes

Outcomes	MMF group (n=85)	SC group (n=85)	Unadjusted HR (95%CI)	*P value*	Adjusted HR * (95%CI)	*P value*
**Secondary Outcome [a]**						
eGFR decline>30%, n (%)	11 (12.9)	30 (35.3)	0.33 (0.16-0.65)	0.002	0.28 (0.13-0.58)	0.001
			**Unadjusted OR (95%CI)**	***P value***	**Adjusted OR * (95%CI)**	***P value***
Rapid renal function decline, n (%)	12 (14.1)	28 (32.9)	0.33 (0.15-0.70)	0.005	0.25 (0.11-0.57)	0.001
			**Group Difference**	***P value***	--	--
Annual rate of eGFR loss, ml/min/1.73m^2 per year	1.2 ± 0.56	3.8 ± 0.57	2.6	<0.001	--	--
Decrease in UPER, % of value at randomization (Q1-Q3)	57.1 (0.0 to 85.1)	28.2 (-60.7 to 52.2)	28.9	<0.001	--	--

Abbreviation: eGFR, estimated glomerular filtration rate; HR, hazard ratio; MMF, mycophenolate mofetil; OR, odds ratio; SC, supportive care; UPER, urinary protein excretion rate.

© 2023 Hou FF et al. *JAMA Network Open.*

eTable 2. Summary of Major Adverse Events

	MMF group (n=85)			SC group (n=85)		p‡
	No. of patients	No. of events		No. of patients	No. of events	
**SAEs** *	4	4		1	1	0.368
**Withdrawn MMF due to AEs**	3	3		--	--	--
**AE of special concerns** †						
**Infections**	14	17		9	10	0.370
Pneumonia	6	8		1	1	--
Upper respiratory tract infection	3	3		3	3	--
Varicella zoster	4	4		1	1	--
Urinary tract infection	1	1		3	3	--
Other infections	1	1		2	2	--
**Gastrointestinal symptoms**	10	11		0	0	0.001
**Elevated serum transaminase**	5	5		1	1	0.210
**Leukopenia**	1	1		0	0	1.00
**New-onset anemia**	7	7		8	8	1.00

* SAEs, serious adverse events, defined according to the International Conference on Harmonization Guideline for Clinical Safety Data Management.
† AE of special concern was defined as drug-related AE listed in the drug information provided by the pharmaceutical manufacture of MMF.
‡ Comparison of proportion of patients between two groups using Fisher exact test.
Abbreviation: MMF, mycophenolate mofetil; SC, supportive care; AE, adverse event.

© 2023 Hou FF et al. *JAMA Network Open.*

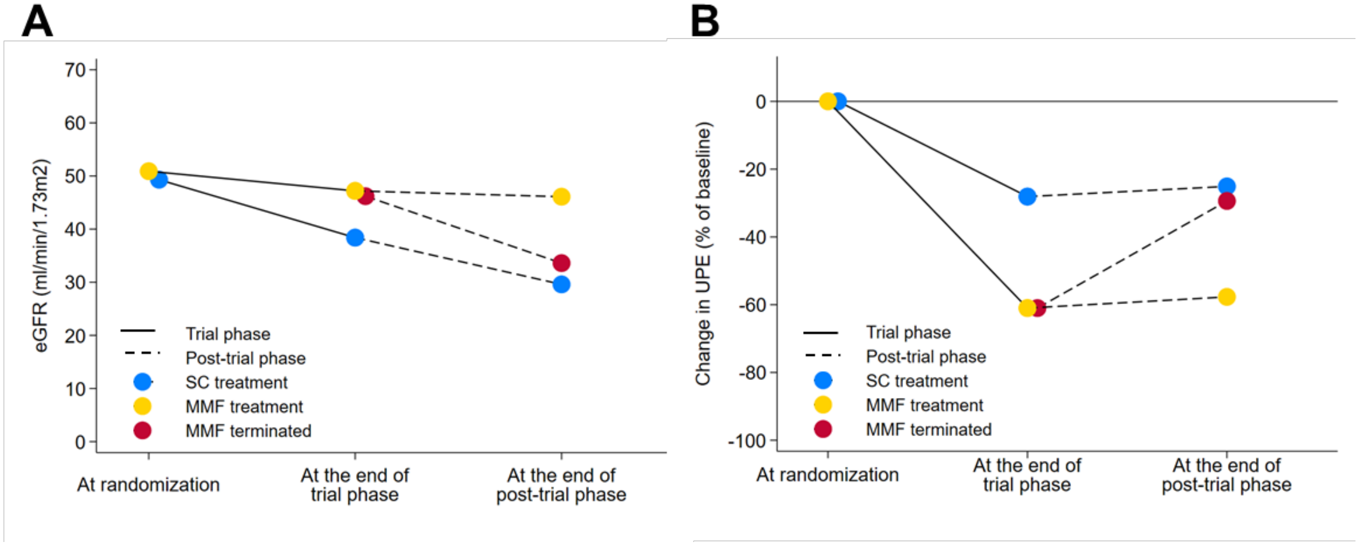

eFigure. Mean Estimated Glomerular Filtration Rate and Changes in Urinary Protein Excretion in Trial and Posttrial Phases

A. Mean eGFR value at randomization, at the end of trial, and at the end of post-trial phase in MMF and SC groups. B. Changes of urinary protein excretion rates from the value at randomization.

Abbreviation: eGFR, estimated glomerular filtration rate; MMF, mycophenolate mofetil; SC, supportive care; UPER, urinary protein excretion rate.
