# Supplementary material for: Effectiveness of Mycophenolate Mofetil Among Patients With Progressive IgA Nephropathy: A Randomized Clinical Trial
Source: JAMA Netw Open. 2023 Feb 6;6(2):e2254054. doi: 10.1001/jamanetworkopen.2022.54054 (PMC12578496; doi:10.1001/jamanetworkopen.2022.54054)
Supplement: Supplement 3. — Group Authors [file jamanetwopen-e2254054-s003.pdf]

*Group Name(s): The MAIN Trial Investigators						
***First Name and Middle Initial(s)**	***Last Name**	***Suffix (eg, Jr, III)**	Academic Degrees	Institution	Location (city, state/province, country)	Role or Contribution, eg, chair, principal investigator
Hao	Zhao		M.D., Ph.D.	Division of Nephrology, Nanfang Hospital, Southern Medical University	Guangzhou, Guangdong, China	Data collection
Xiaolei	Tao		M.D., Ph.D.	Division of Nephrology, Nanfang Hospital, Southern Medical University	Guangzhou, Guangdong, China	Data collection
Yue	Cao		Ph.D.	Division of Nephrology, Nanfang Hospital, Southern Medical University	Guangzhou, Guangdong, China	Statistical analyses
Licong	Su		M.D., Ph.D.	Division of Nephrology, Nanfang Hospital, Southern Medical University	Guangzhou, Guangdong, China	Support with data handling
Yanqin	Li		M.D., Ph.D.	Division of Nephrology, Nanfang Hospital, Southern Medical University	Guangzhou, Guangdong, China	Support with data handling
Shuling	Yuan		B.S.	Division of Nephrology, Nanfang Hospital, Southern Medical University	Guangzhou, Guangdong, China	Data entry and handling
Zhanmei	Zhou		B.S.	Division of Nephrology, Nanfang Hospital, Southern Medical University	Guangzhou, Guangdong, China	Handling pathologic data
Jianwei	Tian		B.S.	Division of Nephrology, Nanfang Hospital, Southern Medical University	Guangzhou, Guangdong, China	Laboratory measurements
Ting	Chen		B.S.	Division of Nephrology, Nanfang Hospital, Southern Medical University	Guangzhou, Guangdong, China	Distributing randomization code and treatment drug
