# Supplementary material for: Effectiveness of Mycophenolate Mofetil Among Patients With Progressive IgA Nephropathy: A Randomized Clinical Trial
Source: JAMA Netw Open. 2023 Feb 6;6(2):e2254054. doi: 10.1001/jamanetworkopen.2022.54054 (PMC12578496; doi:10.1001/jamanetworkopen.2022.54054)
Supplement: Supplement 4. — Data Sharing Statement [file jamanetwopen-e2254054-s004.pdf]

Data Sharing Statement

Hou. Effectiveness of Mycophenolate Mofetil Among Patients With Progressive IgA Nephropathy: A Randomized Clinical Trial. *JAMA Netw Open*. Published online February 6, 2023. doi:10.1001/jamanetworkopen.2022.54054

Data
Data available: Yes
Data types: Deidentified participant data
How to access data: ffhouguangzhou@163.com
When available: With publication

Supporting Documents
Document types: None

Additional Information
Who can access the data: Researchers whose proposed use of the data has been approved.
Types of analyses: For a specified purpose.
Mechanisms of data availability: After approval of a proposal, or with a signed data access agreement.
Any additional restrictions: None.

© 2023 Hou FF et al. *JAMA Network Open*.
